# Holobiont–holobiont interactions across host–ectoparasite systems

**DOI:** 10.1186/s13071-025-07026-0

**Published:** 2025-09-24

**Authors:** Štefánia Skičková, Karolína Svobodová, Myriam Kratou, Alexandra Corduneanu, Ana Laura Cano-Argüelles, Justé Aželytė, Miray Tonk-Rügen, Viktória Majláthová, Dasiel Obregon, Elianne Piloto-Sardiñas, Vaidas Palinauskas, Alejandro Cabezas-Cruz

**Affiliations:** 1 Department of Animal Physiology, Institute of Biology and Ecology, Faculty of Science, Pavol Jozef Šafárik University in Košice, Košice, Slovakia; 2 Faculty of Science, University of South Bohemia, České Budějovice, Czech Republic; 3 Laboratory of Microbiology, National School of Veterinary Medicine of Sidi Thabet, University of Manouba, 2010 Manouba, Tunisia; 4 Department of Parasitology and Parasitic Diseases, University of Agricultural Sciences and Veterinary Medicine, Cluj-Napoca-Napoca, Romania; 5 Department of Animal Breeding and Animal Production, University of Agricultural Sciences and Veterinary Medicine, Cluj-Napoca-Napoca, Romania; 6 Parasitology Laboratory, Institute of Natural Resources and Agrobiology of Salamanca (IRNASA, CSIC), Cordel de Merinas, 40-52, 37008 Salamanca, Spain; 7 State Scientific Research Institute, Nature Research Centre, Akademijos G. 2, Vilnius, Lithuania; 8 Institute for Insect Biotechnology, Justus Liebig University of Giessen, Giessen, Germany; 9 School of Environmental Sciences, University of Guelph, Guelph, ON Canada; 10 Direction of Animal Health, National Center for Animal and Plant Health, Carretera de Tapaste y Autopista Nacional, Apartado Postal 10, 32700 San José de Las Lajas, Mayabeque, Cuba; 11 UMR BIPAR, Laboratoire de Santé Animale, ANSES, INRAE, Ecole Nationale Vétérinaire d’Alfort, 94700 Maisons-Alfort, France

**Keywords:** Holobiont, Microbiome, Host, Ectoparasite, Microbial interactions

## Abstract

Holobionts – hosts together with their resident microorganisms – provide a framework for studying life as a network of interdependent partners. Within host–ectoparasite holobionts, the dialogue between the two microbiomes offers powerful clues to ecological balance, disease dynamics and evolution. Because each holobiont is structurally and functionally compartmentalised, microbes exchanged at the interface can elicit highly local, niche-specific effects that ripple through the system. This review synthesises evidence for microbiota-to-microbiota interactions in four models: *Varroa* mite–honeybee, tick–vertebrate, bat fly–bat and mosquito–vertebrate pairs. In all cases, microbes move passively during feeding or contact, then colonise, replicate and modulate physiology and immunity, exerting a longer-lasting influence than transient biochemical cues. We further introduce the idea of indirect modulation, whereby abiotic or biotic factors act on a recipient holobiont through the intermediary of transferred microbes, underscoring the adaptive plasticity of holobiont networks. Bidirectional cross-talk forms self-reinforcing feedback loops that can redefine a microbe as pathogen, symbiont or immunomodulator, and tune its virulence according to context. These mechanisms shape disease transmission, resistance traits and the overall health of both partners. A deeper grasp of such cross-holobiont dynamics will pave the way for microbiota-based vaccines, targeted microbiome engineering and other innovative tools for human, veterinary and environmental health.

**Figure:**
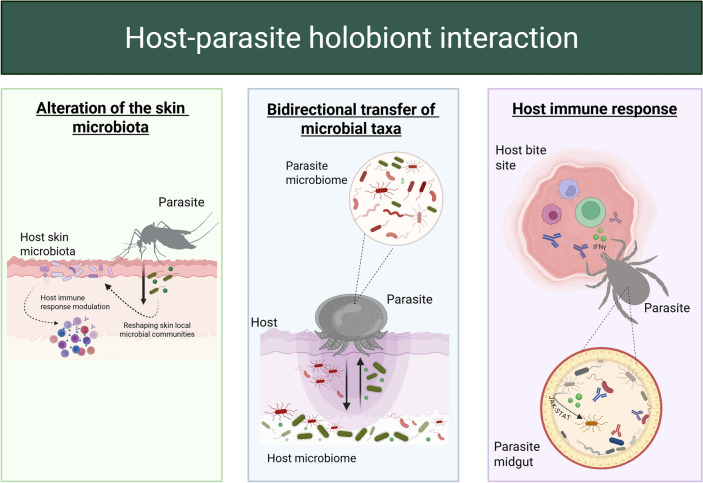

## Background

**Fig. 1:**
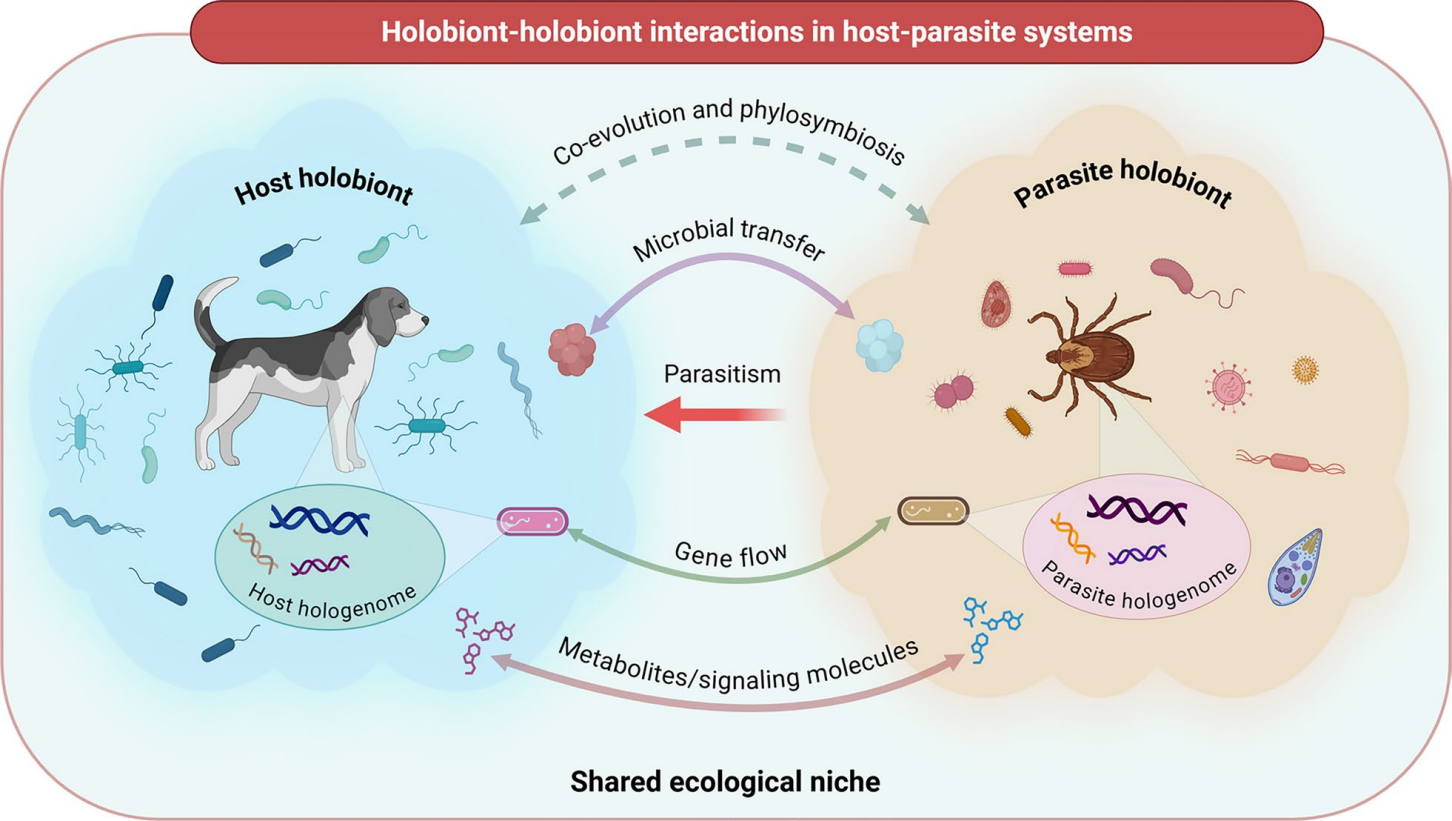
Schematic illustrating the complex interactions between a host holobiont and an ectoparasite holobiont within a shared ecological niche. Each holobiont consists of its host or ectoparasite together with associated microbial communities and their collective genomic material, forming a hologenome. The figure highlights major processes in host–ectoparasite holobiont systems, including parasitism, microbial transfer, gene flow among microbial communities and bidirectional exchange of metabolites and signaling molecules. Co-evolution and phylosymbiosis shape the composition, structure and function of both microbiomes, influencing evolutionary dynamics and biological outcomes for each partner

The notion that animals, plants and even blood‑feeding arthropods live in intimate, evolutionarily stable association with complex microbial consortia has reshaped every branch of the life sciences. The umbrella term holobiont, forged from the Greek holos (whole) and bios (life), defines a macroscopic host together with all of its resident microorganisms as a single ecological and evolutionary unit [1]. Earlier authors treated hosts and microbes as separate actors whose encounters could be analysed with classical parasitology or mutualism theory [2, 3]; however, high‑throughput sequencing, metabolomics and network modelling have revealed a much deeper degree of integration [4–6]. Far from static, each holobiont behaves as a structurally and functionally compartmentalised system whose microbial membership varies with ontogeny, season, diet and abiotic stress, while internal niches such as the gut, cuticle and salivary glands impose micro‑scale selection that sorts taxa according to functional traits [1]. Figure 1 shows this multi‑level architecture for a generic host–ectoparasite pair and highlights the principal routes – microbial transfer, gene flow and exchange of metabolites or signalling molecules – through which partners influence one another.

When two holobionts occupy the same habitat, as happens when a honeybee encounters a *Varroa* mite, a vertebrate hosts a tick, a bat carries a nycteribiid bat fly or a human is bitten by a mosquito, the classical dyad of “host versus parasite” expands into a tripartite ecosystem. Hosts and parasites bring along their own microbiomes, so four living components interact simultaneously inside a shared niche [7]. Across this interface, molecules, cells and entire microbes commute, modulating immunity, metabolism and even behaviour [8–10]. In *Varroa*‑infested colonies, mites deliver deformed‑wing virus (DWV) to bees yet pick up bee‑derived *Gilliamella* and *Snodgrassella* that subsequently shape mite gut physiology and vectorial capacity [11–16]. Ticks feeding on small mammals transmit *Borrelia burgdorferi*, the agent of Lyme borreliosis [17], while simultaneously acquiring host skin commensals whose antioxidant metabolites counter the oxidative burst required for spirochaete colonisation [18, 19]. These bidirectional fluxes mean that each member can remodel its partner’s microbiome and, by extension, its phenotype.

The first layer of cross‑talk is biochemical. Salivary secretions from mites, ticks, bat flies or mosquitoes contain vasodilators, anticoagulants, platelet inhibitors and immunomodulatory peptides that blunt host defences and facilitate prolonged feeding [20–23]. Their influence, however, is spatially and temporally limited: Most of these molecules diffuse only millimetres from the bite site and degrade within minutes to hours. A second, longer‑lasting layer involves the passive transfer of live microorganisms. Once introduced, microbes can establish persistent colonies, participate in metabolic networks and even shift the ecological roles of resident taxa. In mosquitoes, for instance, *Serratia* introduced during probing modifies local cytokine profiles in the vertebrate dermis, while haem‑derived host factors ingested by the insect alter mid‑gut redox status and thereby the mosquito’s competence for *Plasmodium* [24]. Because transferred microbes replicate and disperse within the recipient, their influence routinely eclipses that of transient salivary factors.

Parasite‑associated microbiomes rarely fit neatly into the canonical categories of pathogen, commensal or mutualist. *Rickettsia* offers an instructive illustration. *Rickettsia buchneri* resides obligately in *Ixodes scapularis*, provisioning B vitamins absent from vertebrate blood meals [25]. Closely related *Rickettsia rickettsii* causes Rocky Mountain spotted fever in humans when vectored by *Dermacentor* ticks [26], whereas *Rickettsia peacockii* occupies the same tick species but competitively excludes its pathogenic cousin, thereby lowering disease incidence [27]. Functional plasticity also characterises *Arsenophonus*‑like symbionts of bat flies: Some strains synthesise B vitamins essential for fly development, while others attenuate *Bartonella* proliferation, indirectly benefiting the bat host [28–30]. The ecological role of any given bacterium thus depends on its genome, its compartment and the identity of co‑resident taxa.

No discussion of parasite microbiota would be complete without *Wolbachia*, an α‑proteobacterium that infects an estimated 50% of arthropod species [31, 32]. Inside mosquitoes, it induces cytoplasmic incompatibility, activates innate immune pathways and interferes with multiple RNA viruses. Release programmes on several continents have shown that *Wolbachia*‑infected *Aedes aegypti* populations transmit significantly less dengue (DENV), Zika (ZIKV), and chikungunya virus (CHIKV) than wild‑type counterparts [33, 34]. However, the same capabilities that make this symbiont attractive for vector control entail an inherent biological risk: the possibility that, through adaptive reversions or gene acquisition, it could develop pathogenic traits. This potential change could alter host microbial dynamics and interfere with the establishment of opportunistic infections.

While microbe exchange at vector–host interfaces governs short‑term dynamics, the baseline composition of each holobiont is set by a matrix of abiotic and biotic drivers. Temperature swings restructure tick microbiota and, by extension, their reservoir competence for *Anaplasma* [35]. Humidity and rainfall patterns modulate fungal communities on bat skin, influencing susceptibility to white‑nose syndrome [36]. In both hosts and parasites, sex, developmental stage and diet leave microbial signatures that affect metabolism, reproduction and immunity. These influences feed into a broader eco‑evolutionary process in which natural selection acts simultaneously on host and microbial genomes, shaping the hologenome, a composite genetic repertoire subject to joint evolution [37]. Mounting evidence for phylosymbiosis – the tendency for related hosts to house similar microbial communities – underscores the non‑random nature of microbiota assembly [38, 39].

Internal compartmentalisation further complicates outcomes. In mites, digestive diverticula, Malpighian tubules and cuticular surfaces host bacterial assemblages with divergent metabolic capacities [40, 41]. In vertebrates, the skin microbiome influences volatile organic compounds that mediate mosquito attraction [42]. Consequently, microbial immigrants from another holobiont encounter heterogeneous micro‑environments: some flourish in the gut yet fail on the cuticle and vice versa. The ecological impact of cross‑holobiont transfer is therefore micro‑environment‑specific, reinforcing the idea that regulation is local and context dependent.

Beyond direct transfer, the eco‑holobiont framework embeds host–parasite pairs in wider soil, water and airborne microbial reservoirs. Environmental shifts – pesticide run-off, urban heat islands or climate‑driven habitat change – can alter a donor holobiont’s microbiota, which then shapes a recipient during subsequent encounters, a process termed indirect modulation [43]. For example, insecticide exposure in larval mosquito habitats selects for *Pseudomonas* strains enriched in detoxification genes; adult mosquitoes emerging from such sites show gut communities that, when introduced to vertebrate hosts, tweak cytokine responses and prolong *Plasmodium* development [44]. Similar indirect pathways operate in terrestrial ecosystems where drought reshapes plant root microbiota, altering sap‑feeding insect communities and downstream predator–prey dynamics.

Taken together, biochemical exchange, microbial transfer, environmental modulation and evolutionary feedback build a multidimensional web that controls host health, pathogen transmission and ecological resilience. By analysing four exemplar systems – *Varroa*–honeybee, tick–vertebrate, bat fly–bat and mosquito–vertebrate – we can expose recurrent themes such as compartment‑restricted microbial hotspots, convergence on keystone taxa and feedback‑mediated toggling between pathogenic and mutualistic states. These insights underpin emerging interventions, from keystone‑taxon vaccines in ticks [45–48] to large‑scale *Wolbachia* releases against arboviruses, and they will be critical for forecasting how global change reshapes vector‑borne disease dynamics.

## Case studies of holobiont–holobiont interactions

### *Varroa*-bee system

Western honeybees (*Apis mellifera*) are vital agricultural pollinators, contributing millions of dollars annually to the global economy [49]. Despite their pivotal role in food production, honeybee colonies have faced significant declines in recent decades [50–52]. One major driver of these losses is the infestation by *Varroa destructor* mites, specialised ectoparasites that feed on fat body and haemolymph of honeybees, and vector of various pathogenic microorganisms, severely compromising honeybee physiology [53].

Considering holobionts as structurally and functionally dynamic units implies that their taxonomic composition and microbial assemblage are subject to variability influenced by biotic and abiotic ecological determinants. However, like any system, they must maintain a certain level of stability, which is why they are characterised by the existence of a conserved microbial core, generally resistant to external perturbations. Corresponding to this, the honeybee gut microbiota has been show to consist of core bacterial taxa, including *Lactobacillus* (Firm-4 and Firm-5), *Bifidobacterium*, *Snodgrassella alvi*, *Gilliamella apicola* and *Frischella perrara*, as well as *Bartonella apis*, *Bombella apis* and *Commensalibacter* [12]. These bacteria play a nutritive role, promote detoxification of dietary compounds and pesticides and provide protection against pathogens by inhibiting harmful microbes, such as *Paenibacillus larvae* [13], through metabolic and immune-supportive functions [14–16]. The successful completion of the core’s essential functions within the system demonstrates a successful process of microbial interaction. From this, one can hypothesise that, while ecological competition may exist owing to exploitation or interference, what prevails within the microbial core of the holobiont are synergistic and mutualistic interactions designed to maintain systemic stability. Overall, the absence of antagonistic relationships within the core suggest a stable ecological configuration with a prevalence of potentially mutualistic and functionally complementary interactions.

Interestingly, these genera can also be found in the microbiome of *Varroa* mites, which confirms that honeybees and their infesting *Varroa* mites share common bacterial taxa [54, 55]. The presence of common bacterial genera in the microbiomes of both systems suggests possible microbial transfer during prolonged contact at the permissive honeybees–*Varroa* mites interface. This phenomenon implies the absence of colonisation resistance and, consequently, the establishment of the transferred microorganisms in the mite and the possible formation of functional microbial cores. Community reconfiguration occurs, leading to ecological stability based on possible mutualistic interactions as cross-feeding or syntrophy. In this context, the holobiont maintains a stable microenvironment that allows for microbial co-evolution (Table 1).

**Table 1:** Synoptic comparison of *direct* and *indirect* microbiota cross-talk in four host–ectoparasite holobiont systems

Host ↔ ectoparasite pair	Physical interface and major transfer route(s)	Direct microbial transfer (examples and direction)	Indirect interactions (molecule/process and direction)	Functional impact on host holobiont	Functional impact on ectoparasite holobiont	Key references
Honeybee (*Apis mellifera*) ↔ Varroa destructor mite	Feeding wounds on cuticle; reciprocal contact inside hive	• *Gilliamella*, *Lactobacillus*, *Snodgrassella* bee → mite • Deformed-wing virus (DWV) and *Paenibacillus larvae* mite → bee	• Bee-derived AMPs (apidaecin, abaecin) primed by core gut taxa bee → mite • *Paenibacillus* secondary metabolites remodel local bacterial networks within bee compartments mite → bee	• AMPs and probiotic-like core taxa curb DWV and *P. larvae* virulence; maintain gut homeostasis	• Acquisition of bee bacteria forms a stable gut core and influences mite fitness and vectorial capacity	[54, 56–64]
Tick (*Ixodes spp.*) ↔ vertebrate host (mouse/human)	Skin penetration and prolonged blood-meal; ingestion of host serum molecules	• *B. burgdorferi* tick → host • Skin commensals (Lachnospiraceae, Muribaculaceae) host → tick	• Host IFN-*γ* and α-Gal/anti-microbiota antibodies host → tick • Tick salivary immunomodulators tick → host	• Local microbiome depletion plus saliva factors facilitate spirochaete entry; antibodies lower pathogen burden	• Host IFN-*γ* triggers JAK/STAT-IGTPase → Dae2 axis, killing *Borrelia*; antibodies reshape midgut community and raise mortality	[45, 46, 73, 83–85, 91, 98]
Bat ↔ bat fly (Nycteribiidae/Streblidae)	Continuous skin/fur contact, blood-feeding, social grooming	• *Bartonella* spp. bat → bat fly (and vice versa) • Arsenophonus-like endosymbionts bat fly → bat	• B-vitamin provisioning by Arsenophonus to bat fly (fly → bat fly) enhances fly survival • Social grooming and co-roosting drive colony-level microbial seeding bat → bat fly	• Zoonotic *Bartonella* spill-over risk; nutrient loss to fly may affect host condition	• Vitamin symbiosis boosts fly fecundity; bat immune cues likely filter fly microbiome	[28–30]
Mosquito (*Anopheles/Aedes spp.*) ↔ vertebrate host (human/bird)	Repeated blood meals; salivary probing	• *Serratia* and *Elizabethkingia* mosquito → host bite site • Skin/gut commensals host → mosquito midgut	• Host gsNAbs (anti-α-Gal) and cytokines (TGF-β1, IFN-*γ*, TNF-α) host → mosquito; activate JAK-STAT or MEK-ERK-NOS pathways • Peritrophic matrix and IMPer barrier mosquito → host-derived microbes	• Local inflammation/dysbiosis can raise or lower susceptibility to arboviruses/malaria	• Host antibodies and cytokines disrupt gut microbiota, boost AMPs/NO, cut Plasmodium load and lower vector competence	[73, 93, 133, 134, 138–141]

The research conducted by Skičková et al. [54] revealed that the keystone in the microbiomes of honeybees and *Varroa* mites infected by *Paenibacillus* spp. closely align with the findings of Moran [12]. In the microbiome of honeybees highly infected with *Paenibacillus*, the keystone taxon was *Bifidobacterium*, whereas in mites with low *Paenibacillus* loads, members of the Orbaceae family were predominant. In honeybees with low *Paenibacillus* infection, the keystone taxa were *Lactobacillus* and *Gilliamella*, while in lowly infected *Varroa*, it was *Snodgrassella* [54]. These findings suggest that while *Paenibacillus* spp. can proliferate on both honeybee and *Varroa* holobionts, each system provides an ecological environment that shapes microbial interactions differently. Furthermore, the passive nature of bacterial transfer to the mite likely results in a low inoculum density, allowing for greater colonisation resistance and a lower microbial load. As a result, the microbiome in mites remains relatively stable, and the emerging social microbial dynamics differ from those in the honeybee. This differentiation suggests that similar microbial taxa can behave differently depending on specific host microbial architectures and ecological pressures, reflecting a nuanced pattern of holobiont–holobiont interactions.

The study also identified the positive and negative network associations, on the basis of eigenvector centrality, between *Paenibacillus* and other bacterial genera. In highly infected honeybee networks, *Paenibacillus* was positively linked with three genera: *Enterococcus*, *Bifidobacterium*, and *Lactobacilllus*, and negatively associated with *Spiroplasma*. However, in *Varroa* mites highly infected with *Paenibacillus*, *Paenibacillus* was only negatively associated with *Pseudomonas* [54]. Since various *Paenibacillus* species produce secondary metabolites (e.g. polymyxins, polyketides and paenilarvins), which possess antimicrobial and antifungal effects [56, 57], it is suggested that these compounds could suppress the growth of certain bacterial species that might form interactions within the microbiome. The production of such bioactive compounds can contribute to maintaining a balanced microbial environment by inhibiting the colonisation of potentially pathogenic or competitive bacteria [58]. The antimicrobial activity of *Paenibacillus* restructures the community and interferes with the colonisation of microorganisms, but it does so within a localised context in restricted compartments of the holobiont. This concentration of activity contributes to the ecological stability of the system, as microbial taxa operate within defined microenvironments, preventing the destabilisation of functionally specific microbial cores.

**Fig. 2:**
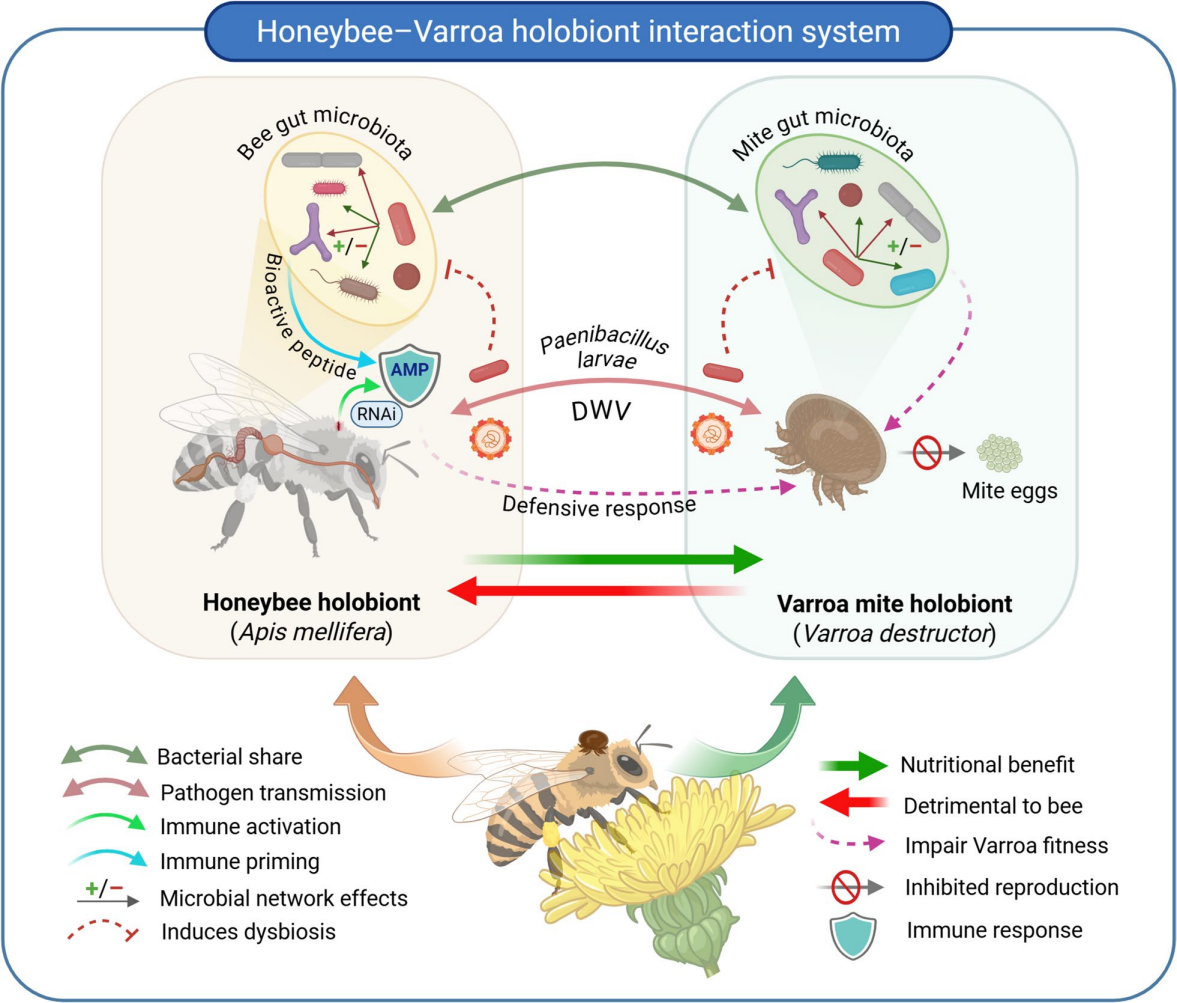
Schematic illustrating the multifaceted interactions between the honeybee holobiont (*Apis mellifera*) and the mite holobiont (*Varroa destructor*). Each holobiont consists of its respective host or ectoparasite, and associated gut microbiota, with microbial networks that influence and respond to each other through multiple mechanisms. Bacterial sharing reflects overlapping core microbiota taxa, while pathogen transmission, such as with deformed wing virus (DWV), is facilitated by the mite, contributing to honeybee morbidity. The potential involvement of *P. larvae* is shown for conceptual completeness; however, current evidence does not confirm direct transmission from *Varroa* to honeybees. *Varroa* feeding triggers immune activation in the bee (green arrows), including upregulation of antimicrobial peptides (AMPs) and ribonucleic acid interference (RNAi). The bee’s microbiota also plays a role in immune priming and pathogen suppression. Both *Paenibacillus* and DWV can induce dysbiosis (dashed arrows), disrupting the microbiome balance within either holobiont. Microbial network effects (indicated as +/−) illustrate complex intra-holobiont microbial interactions. Bee defensive responses (immune- and microbiome-mediated) are hypothesized to impair *Varroa* fitness and reproduction; however, these effects require further experimental validation. Nevertheless, the predominant outcome remains detrimental to the honeybee and nutritionally beneficial to the mite. The system exemplifies a dynamic, bidirectional holobiont–holobiont interaction, influencing host and parasite fitness, immunity, and disease susceptibility

Although the interactions between *Paenibacillus* and the adult honeybee microbiota have not been extensively studied, Truong et al. demonstrated that certain *Lactobacillus* species inhibit the growth of *P. larvae* and reduce its harmful effects in honeybee larvae [59]. Honeybee *Lactobacillus* species and other lactic acid bacteria produce several bioactive compounds that can contribute to maintaining a balanced microbial environment by inhibiting the colonisation of potentially pathogenic or competitive bacteria [60]. Limiting the growth of some bacteria, especially pathogens, helps promote the survival of beneficial microorganisms, thus enhancing host health. In addition, the antimicrobial and probiotic effects of some core bacterial strains have an impact on broader microbial community, influencing the dynamics of microbial populations and their interactions with the host. This ecological role of secondary metabolites may also contribute to shaping the resilience of the microbiome to environmental stressors or pathogen challenges (Fig. 2) [59]. The ecological relevance and keystone taxon status of *Lactobacillus* in the honeybee microbiome suggest the possibility of passive transfer to *Varroa* destructor during close contact at the bee–mite interface. Once inside the mite, these bacteria could modulate the microbial composition of *Varroa*, either transiently or through more stable colonisation, thereby influencing the structure of its microbiome and possibly its vectorial capacity. Such microbial transfer represents a potential mechanism of holobiont-to-holobiont cross-talk, with implications for the evolution of symbiotic relationships and the development of biological control strategies targeting vector-associated microbiomes.

*Varroa* mite infestation and associated pathogens trigger extensive transcriptional changes in honeybees [61–63]. These changes include upregulation of immune system genes coding for antimicrobial peptides (AMPs), RNA interference (RNAi) machinery, and recognition molecules [61–64], which can also affect the honeybee gut microbiota. Some studies report a significant increase in gut microbial diversity following *Varroa* infestation [65], while others found no remarkable differences (Table 1) [66].

*Varroa* mite has become an efficient vector of several deadly honeybee viruses, including deformed wing virus (DWV), which is one of the most significant threats to *A. mellifera* honeybees (Table 1). Pathogenicity can be triggered in bees even with naturally low viral loads by some bacterial co-infections. For example, honeybees artificially infected with *Escherichia coli* exhibited increased DWV titers and developed deformed wings [67]. However, honeybees mono-inoculated with *S. alvi* or *G. apicola* demonstrated enhanced bacterial clearance from haemolymph following *E. coli* injection and exhibited higher levels of AMPs compared with microbiome-free bees, suggesting immune priming by these core gut community members [68]. Similarly, *F. perrara* induced immune priming by enhancing the production of the specific antimicrobial peptide apidaecin [16].

Feeding wounds on honeybee’s body caused by *Varroa* mites feeding often facilitate bacterial infiltration [69], potentially exacerbating viral infections. This can lead to dysbiosis, or a microbial imbalance, further increasing pathogen susceptibility. Tetracycline-induced dysbiosis in honeybees has been shown to promote infiltration by opportunistic bacteria and reduce lifespan following infection with *Serratia marcescens* pathogen [70]. Similarly, stress-induced microbiota alternations in honeybees have been linked with heightened susceptibility to *Lotmaria passim* [71] and worsened outcomes of DWV infections [72].

Understanding the intricate interactions within the honeybee holobiont, including *Varroa* mites, associated viruses, and other microbial communities, is essential for developing effective strategies to combat honeybee colony losses and ensure sustainable pollination services.

### Tick–host system

Ticks are obligate hematophagous ectoparasites of terrestrial vertebrates and serve as efficient vectors of a wide range of pathogens. The colonisation, establishment and subsequent transmission of pathogens by ticks are influenced by the behaviour and composition of their vector-associated microbial communities. These cooperative or competitive interactions can directly modulate pathogen dynamics within the vector. Processes such as the production of antimicrobial compounds, signaling mechanisms or the synthesis of secondary metabolites that serve as precursors to essential metabolic pathways can facilitate pathogen development or favour the establishment of other microorganisms. Together, these microbial interactions create a permissive environment that promotes pathogen persistence and enhances transmission efficiency [73, 74].

A bidirectional crosstalk is established between the tick microbiota and that of its vertebrate host, both considered holobionts (Table 1). During hematophagous feeding, the tick can acquire microorganisms from the host’s skin, metabolites, cytokines, extracellular vesicles and other immunological factors that can reconfigure its own microbiome in terms of composition, diversity and assembly. This dynamic exchange at the holobiont level suggests that the host microbiota could play an active role in pathogen colonisation and subsequent transmission, and thus in shaping vector competence, adding a new layer of complexity to the ecology of tick-borne diseases [47, 73].

The tick functions as a compartmentalised ecosystem where tissues such as the midgut, salivary glands and ovaries constitute micro-environments with specific microbial compositions and community assembly. This compartmentalisation implies that the host influence is differential in each niche, depending on acquired components such as the cutaneous microbiota, metabolites, extracellular vesicles and cytokines. The midgut, as the primary interface for host-derived components, can accentuate phenomena such as resistance to pathogen colonisation. In this context, the resident microbiota prevents the establishment of exogenous microorganisms or the overgrowth of native taxa through metabolic competition, the production of antimicrobial compounds and immune activation [73]. Consequently, the tick will function as an integrated and dynamic ecological unit where interactions between the vector, its microbiota and external stimuli (such as those derived from the host) shape its functionality and vector competence.

The conceptualisation of the holobiont framework, which integrates the host and its microbiome as a cohesive biological entity [75], has further evolved into the pathobiome paradigm, emphasising the role of microbial consortia in disease ecology, including tick-borne infections [76, 77]. Therefore, elucidating these microbe–microbe interactions is crucial for understanding their broader implications on tick physiology and vectorial capacity [45, 78, 79].

Within this framework, the host must be considered a biologically complex holobiont with considerable microbial dynamics. Before any interaction with a tick, the host holobiont is influenced by diverse and successive microbial exposures from interactions with the environment, co-infestations with other ectoparasites, the resident microbiota and contact with other hosts. This complex microbial landscape not only modulates the host’s immune and metabolic systems but also influences the response of its microbiota to vector-mediated perturbations during cross-talk. In this context, the host’s contribution to cross-talk goes far beyond passive reception; it actively conditions the nature of microbial exchange and pathogen dynamics at the tick-host interface. For instance, Boulanger et al. observed cross-talk between the *Ixodes ricinus* tick microbiome and mouse skin microbiome, while at the site of the tick bite, the skin microbiome was entirely altered [80].

The skin is the first physical and immunological barrier against antigens, including ectoparasites such as ticks. The host skin microbiota plays a critical role in shaping host–tick–pathogen interactions and providing surface for dynamic microbial interference, where the tick may acquire microorganisms as it feeds. This interface can be considered a starting point for understanding holobiont–holobiont interaction chains between host, tick, and pathogen [81, 82]. Notably, recent research by Boulanger et al. demonstrated that tick blood-feeding induces a profound alterations of the host skin microbiome [80]. Several bacterial families, such as Lachnospiraceae and Muribaculaceae, which are typically associated with beneficial functions in the skin and gut of mice and humans [83–85], were significantly depleted following tick attachment (Table 1). Several studies have explored the relationship between *Ixodes* spp. and their hosts, particularly focusing on the host’s influence over tick microbiomes. While findings suggest that the tick species itself is the primary determinant of microbiome composition, with minimal correlation to host microbiota, some nuances exist [86–88]. For example, *I. scapularis* and *Dermacentor variabilis* microbiomes remained distinct regardless of feeding on prairie voles or white-footed mice [86]. Nonetheless, other research indicates that host species can still modulate the overall microbiome composition in ticks, influencing the relative abundance of specific bacteria such as *R. buchneri* [89].

To further elaborate this “continuum”, during blood feeding, hematophagous vectors such as mosquitoes and ticks ingest host-derived molecules, including antibodies [46, 48, 90] and cytokines [91–93]. These host antibodies can interact directly with the vector gut microbiota, while cytokines influence vector immune responses. This concept is supported by experimental evidence from microbiota-targeting vaccines, which demonstrate that host antibodies can alter vector microbiome composition [46], impair vector fitness [45, 94] and reduce vector competence [48, 90]. Indeed, host immunisation with keystone taxa from the tick microbiota (highly connected microbes that shape community structure and function) induces bacterial-specific antibodies that significantly increase tick mortality [45]. These keystone taxa, widely present in the tick microbiota, likely play essential roles in microbial network stability and tick physiology [74, 78, 95]. Their targeting by host immune responses suggests a direct link between vertebrate immunity and microbiota dynamics in the tick holobiont.

A key example is the antibody response against galactose-α−1,3-galactose (α-Gal), a carbohydrate synthesised by microbial galactosyltransferases within the host and tick microbiotas. Host antibodies against α-Gal contribute to tick mortality upon blood feeding, demonstrating how vertebrate immune factors disrupt microbial-tick associations (Table 1) [45]. Beyond direct microbial targeting, host-derived antibodies also interact with tick tissues and intracellular proteins once ingested. For instance, antibodies against the protective tick antigen Bm86 – a glycoprotein located in the gut epithelial cells [96] – bind to intestinal cell surfaces, causing cell lysis and reducing female reproductive success [97].

Recent advances in anti-microbiota vaccines have demonstrated their ability to selectively target key bacterial taxa within the tick microbiota, altering microbial composition and affecting tick fitness [45, 46]. Whereas *Escherichia*-*Shigella* was identified as a central component of tick microbial communities and Enterobacteriaceae family was found to be a keystone taxon, the *Escherichia coli* bacterium, fulfilling both roles, was targeted through host immunisation [45]. Mice immunised with live *E. coli* developed high titers of *E. coli*-specific immunoglobulin (Ig) M and IgG, which negatively correlated with *Escherichia*–*Shigella* abundance in ticks [46]. Ticks feeding on *E. coli*-immunised mice exhibited significantly increased engorgement weight compared with those feeding on mock-immunised controls, highlighting immunisation-driven microbiota modulation [45, 46]. Moreover, high tick mortality was observed when feeding on hosts with elevated IgM and IgG levels against α-Gal [45].

Ticks *I. scapularis*, feeding on mammalian hosts infected with the Lyme disease-causing bacteria *B. burgdorferi*, responded to the interferon *γ* (IFN-γ) cytokine derived from the hosts. The ticks ingesting IFN-γ during feeding showed enhanced expression of the signaling transducer activator of transcription (STAT) factor, which is necessary for activating inducible guanosine triphosphatase (IGTPase). This activation induced the production of antimicrobial peptide Dae2, which helped to kill the *Borrelia* pathogen, resulting in a significant reduction in the *B. burgdorferi* survival in the ticks [73, 91]. Similarly, in another experiment, the ingestion of natural antibodies reduced the spirochete *B. burgdorferi* burden within feeding *I. scapularis* nymphs [98].

This immunological-microbial interplay extends to pathogen control (Table 1). Host antibodies can persist in the tick midgut for varying durations, influencing both microbiota composition and pathogen transmission [99]. Empirical evidence shows that vertebrate antibodies can target vector-borne pathogens within ticks [100]. A notable example is *B. burgdorferi*, where anti-BBA52 antibodies, directed against an outer membrane protein preferentially expressed in feeding ticks, block spirochete transmission to murine hosts without reducing bacterial loads in the tick gut [100].

These intricate holobiont–holobiont interactions between the host and tick microbiotas are pivotal in shaping vector competence, pathogen transmission and overall tick fitness. The reciprocal influence of host immune responses on tick microbial communities, combined with the impact of tick microbiota on host–pathogen dynamics, emphasises the interconnectedness of both holobionts. Understanding these bidirectional interactions opens avenues for targeted interventions, such as anti-microbiota vaccines, that could disrupt this loop, potentially reducing tick-borne pathogen transmission.

### Bat fly–bat system

Bat species are important reservoirs for a wide range of pathogens and are reported to harbour a greater diversity of viruses per species than rodents [101, 102]. Among many ectoparasites that infest bats are ticks (Acari: Argasidae and Ixodidae), mites (Mesostigmata: Spinturnicidae and Macronyssidae), fleas (Siphonaptera: Ischnopsyllidae), bugs (Hemiptera: Cimicidae and Polyctenidae), earwigs (Dermaptera: Arixeniidae) and, most notably, bat flies (Diptera: Nycteribiidae and Streblidae), which represent the most prevalent and specialised group of bat ectoparasites [103, 104].

Bat flies exhibit a blood-feeding dietary strategy, remaining on their bat hosts throughout their entire lives [105]. They exhibit remarkable morphological and behavioural adaptations well-suited to their parasitic lifestyle. Bat flies frequently experience co-infestation, where multiple ectoparasite species inhabit the same host simultaneously, facilitating the exchange of microorganisms within diverse microbiomes. These coexistences generate a dynamic microbial interface between holobionts, enabling interspecific microbial exchange and modulating immunological and metabolic responses in both the vector and the host. These processes exemplify a broader holobiont–holobiont dynamic, similar to that previously described in the tick–host system, where successive microbial exposures from environmental sources or other ectoparasites shape the host’s immune and metabolic landscape prior to any interaction with a specific vector’s microbiota (Table 1).

**Fig. 3:**
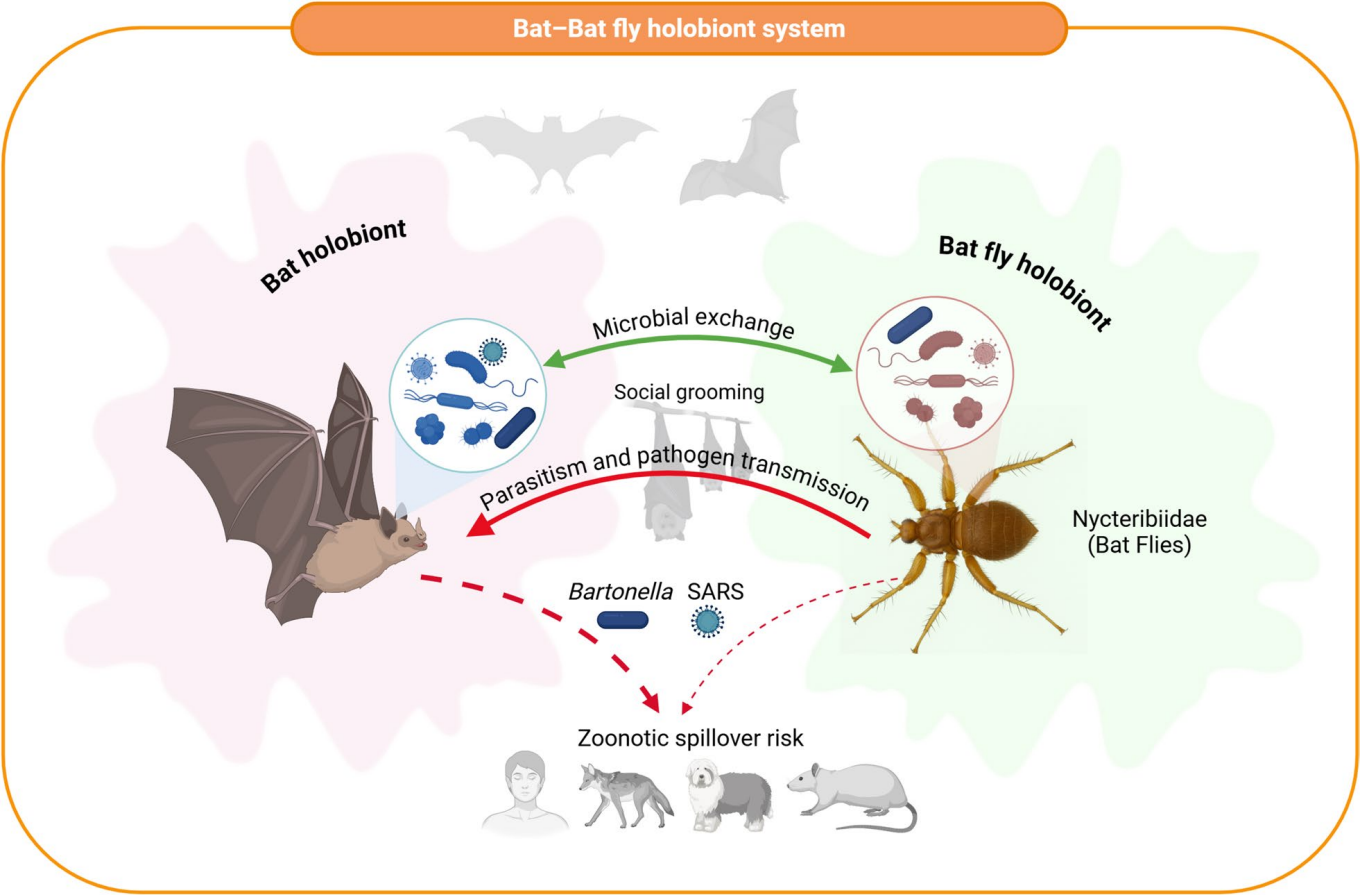
Schematic illustrating the interactions between a bat holobiont and a Nycteribiidae bat fly holobiont. Each holobiont consists of its respective host or ectoparasite along with associated microbial communities. Microbial exchange occurs between bats and their bat flies, facilitated by close physical contact and social grooming behaviours within bat colonies (silhouetted above). Parasitism and pathogen transmission pathways are illustrated by red arrows, highlighting the movement of key zoonotic agents, such as *Bartonella* (bacterium) and severe acute respiratory syndrome (SARS)-like coronaviruses (virus), between bat flies, bats and potential secondary mammalian hosts. The diagram highlights zoonotic spillover risk to humans, domestic dogs and rodents, representing primary exposure routes for these pathogens. This conceptual model underscores the ecological complexity of bat–bat fly holobiont systems and their importance for One Health frameworks and emerging infectious disease surveillance

Additionally, bat hosts living in colonies further promote microbial transmission through social behaviours such as grooming, which influences the spatial distribution and ecological niche specialisation of bat flies (Fig. 3) [106]. These collective behaviours significantly increase pre-exposure to diverse microbial consortia, both in composition and assembly, which reshapes the bat microbiome before any direct interaction with the vector. A directed crosstalk arises between host and ectoparasite, where bat-derived microbial and immunological signals can actively modulate the structure and function of the microbial community within the bat fly holobiont.

Owing to their diverse feeding guilds (insectivores, frugivorous, nectarivores, carnivores and sanguivorous diets) [107, 108], bats can act as vectors for pathogen transmission to other organisms, including humans, creating complex quadripartite interactions. In Nigeria, for example, people enter caves to capture fruit bats (*Rousettus aegyptiacus*) infested by bat-associated nycteribiid flies (*Eucampsipoda africana*); the bats are then prepared and consumed during a traditional festival [109]. Additionally, bat faeces are frequently collected and used as fertiliser for vegetable farms [110]. Notably, individuals engaged in these activities have been found to be seropositive for *Candidatus* Bartonella rousetti, suggesting potential zoonotic transmission route via bat flies [109]. Moreover, another study identified bat flies as potential vector of *Bartonella* spp., capable of transmitting the bacteria to new host organisms [111], such as rodents, carnivores and herbivores, in which *Bartonella* had already been detected [112].

Overall, these findings suggest that bats, ectoparasites and other hosts form a dynamic network of microbial exchange, where each organism functions as an integrated unit with its microbiota. The potential transfer of microbial taxa between bat flies and vertebrate hosts exemplifies a holobiont interaction, in which microbial communities are not only transmitted but can also adapt and reconfigure in new ecological niches, influencing the physiology, immunity and pathogen susceptibility of the new host.

Some *Bartonella* species are known to cause human diseases, including trench fever (*Bartonella quintana*), cat scratch disease (*Bartonella henselae*), Carrion’s disease (*Bartonella baciliformis*) and various forms of bacteraemia or chronic infections (*Bartonella elizabethae*) [113]. Interestingly, similar *Bartonella* genotypes have been identified in the bats from Georgia, sera samples from the forest workers in Poland, and dogs in Thailand [114]. Likewise, *Candidatus* Bartonella mayotimonensis was detected in the aortic valve of a patient with endocarditis in the USA as well as in bats of *Myotis lucifugus* and *Myotis grisescens* [111] (Table 1).

To date, research on the bat fly microbiome remains limited, with most studies focusing on two bacterial groups: *Bartonella* and *Arsenophonus*-like organisms (ALOs), which include species such as *Arsenophonus*, *Aschnera* and *Riesia*. *Bartonella*, an intracellular parasitic bacterium linked to zoonotic diseases [28] has also been detected in ticks (Argasidae: *Ornithodoros* spp., *Carios* spp.) [115, 116], suggesting the potential for co-infection or interactions between bacterial species. In contrast, ALOs are primary endosymbiotic microorganisms that provide essential nutrients, such as vitamin B, to their bat-fly hosts, as the vertebrate blood they feed on is deficient in this nutrient. While *Bartonella* is thought to have co-evolved with bats and bat flies [118], interactions between ALOs and their hosts – bat flies – remain undetected (Table 1) [119].

Bats are often linked to the transmission of various zoonotic diseases to humans, including rabies [120], Ebola haemorrhagic fever [121], Nipah viral encephalitis [122] and numerous species of coronaviruses [140], such as severe acute respiratory syndrome (SARS) [124] and, most recently, the coronavirus disease 2019 (COVID-19) virus [125]. Even ectoparasites, such as bat flies, can harbour various virus species, with the most dominant being narmaviruses, reoviruses and sebemo-like viruses [123].

Analysing vector–host holobionts through the perspective of both global interaction and compartmentalisation allows for a broader interpretation of microbial interactions among microorganisms of different aetiologies and pathogenesis mechanisms. In this context, considering microbial tropism, the dynamic interaction between microorganisms such as viruses and bacteria can be understood through their coordinated influence on colonisation, persistence and transmission. These microbial agents can form complex virus–bacteria associations, where they interact in ways that can influence their survival, replication and transmission. For example, certain bacteria may enhance viral stability or facilitate infection by weakening the host’s immune response. Conversely, some viruses can alter bacterial behaviour, increasing their pathogenicity or enabling co-infections. Such interactions can occur within a shared host, such as bats or their ectoparasites, potentially amplifying the risk of cross-species transmission to humans. Understanding these complex relationships is crucial for evaluating the dynamics of zoonotic disease emergence and developing effective control strategies.

From a holobiont–holobiont perspective, the bat–bat fly system exemplifies a biologically integrated network of microbial community exchange, reconfiguration and co-evolution. Microbiome-level interactions between bats and their ectoparasites are characterised as bidirectional, mediated by shared environments, feeding behaviours and social dynamics. This framework highlights the importance of considering zoonotic emergence not only from the perspective of pathogen spillover but also as a product of microbial co-evolution and ecological connectivity at the holobiont level.

### Mosquito–host system

Mosquitoes are primary vectors of some of the world’s deadliest diseases, including malaria, dengue and lymphatic filariasis [124, 125]. According to the World Health Organization (WHO) [126], malaria alone causes over 650,000 deaths annually, primarily in tropical and subtropical regions. While traditional research has focused on direct pathogen–vector interactions, particularly mosquito physiology, immunity and the parasite–host relationship, recent evidence suggests that these interactions occur within a broader ecological and microbial framework.

Both mosquitoes and their vertebrate hosts function as interconnected holobionts, where the microbiota plays a fundamental role in immune modulation, pathogen transmission and vector competence [127]. Guéran et al. emphasised that an arthropod vector should no longer be viewed as an isolated organism but rather as an interactive system (vector holobiont) in which the vector and its microbiota operate as an integrated unit [128]. While this perspective has shifted research focus towards understanding the influence of mosquito microbiome on pathogen transmission, we propose that the holobiont model must be further expanded – to include the impact of vertebrate host microbiota and immune factors on mosquitoes.

The mosquito microbiota, residing in the gut, salivary glands and reproductive organs, plays a crucial role in shaping vector fitness, immune responses and pathogen susceptibility (Table 1) [129]. Several studies have identified core bacterial taxa shared across mosquito species, while others exhibit species- and organ-specific microbial compositions. Mancini et al. analysed nine mosquito species from the genera *Anopheles*, *Aedes* and *Culex* and found that *Serratia* was present in the salivary glands of all species [130]. Other shared bacterial taxa included *Escherichia*–*Shigella*, *Pantoea*, *Acetobacter*, *Sphingomonas*, *Burkholderia* and *Cupriavidus*. Accoti et al. identified Proteobacteria and Bacteroidetes as dominant phyla in *Anopheles gambiae* and *Anopheles stephensi* microbiota, with *Serratia*, *Elizabethkingia*, *Acinetobacter* and *Comamonas* as the most abundant midgut genera [131]. Onyango et al. reported that *Aedes albopictus* midgut and saliva contain Bacteroidetes and Proteobacteria as core phyla, along with *Elizabethkingia*, *Pseudomonas*, *Sphingomonas* and *Wolbachia* [132].

When mosquitoes take a blood meal, they ingest not only pathogens but also vertebrate immune molecules and microbiota, which can alter their gut microbial community and immune responses. Hematophagous insects, including *Anopheles* mosquitoes, have evolved mechanisms to regulate interactions between ingested blood, gut microbiota and immune responses. One such mechanism is the formation of the peritrophic matrix, a chitinous, semi-permeable barrier that encapsulates the blood meal, preventing direct contact between blood components and the gut epithelium (Table 1) [133]. In addition, the immunomodulatory peroxidase (IMPer), secreted by midgut epithelial cells, cross-links mucin proteins to further limit permeability to immune elicitors and protect resident microbiota from immune attack [134]. Despite these protective barriers, host-derived immune molecules, including cytokines and antibodies, can persist in mosquitoes post feeding. Studies have detected vertebrate host antibodies adhering to the midgut epithelium and even present in the haemocoel of *Anopheles* and *Culex* mosquitoes [135, 136]. Interestingly, Meyers et al. demonstrated that IgG translocation varies across mosquito species, with anti-glutamate-gated chloride channel (anti-GluCl) IgG detected in *An. gambiae* but not in *Ae. aegypti* or *Culex tarsalis*, suggesting species-specific differences in midgut permeability to host immune factors [137].

The peritrophic matrix is suggested to function not only as a barrier for immune elicitors but also contributes to the selective retention of symbiotic and commensal bacteria, allowing certain bacterial species to persist within the midgut while excluding others [138]. This selective filtering function may influence how host-derived microbiota interacts with resident mosquito microbiota, potentially reshaping microbial composition after each blood meal. The presence of host-derived immunoglobulins raises intriguing questions regarding their impact on mosquito physiology, microbiota composition and vector competence (Table 1). Recent findings by Aželytė et al. indicate that natural antibodies, such as anti-α-Gal IgY, can shape mosquito gut microbiota. Their study showed that *Culex pipiens* mosquitoes feeding on birds immunised against *E. coli* O86:B7 (which expresses high levels of α-Gal) exhibited altered microbiota composition. This shift in microbial diversity was associated with reduced *Plasmodium* development, suggesting that host-derived antibodies can influence mosquito microbiota and indirectly modulate vector competence [90].

Similarly, transforming growth factor-*β*1 (TGF-β1), a cytokine present in mammalian blood, remains active post ingestion and triggers MEK-ERK signaling in mosquito cells (Table 1) [139]. Orthologous TGF-β receptors and Smad signaling proteins have been identified in *Anopheles* mosquitoes, indicating that vertebrate immune cytokines may actively regulate mosquito immune responses [93, 140]. This highlights the concept that mosquito immunity is not only shaped by its microbiome but also by vertebrate immune factors [73], reinforcing the cross-talk holobiont concept.

The potential impact of these immune molecules on mosquito microbiota remains an emerging area of research. Cytokines, such as TGF-β1, may influence the balance of gut microbial species by modifying the physiological conditions of the midgut, such as pH levels, antimicrobial peptide production or epithelial integrity [138]. These indirect effects could promote the growth of specific bacterial taxa while suppressing others, thereby altering microbial interactions that influence pathogen survival within the mosquito [141]. Future studies should investigate whether host immune molecules can persist within mosquito tissues long enough to have lasting effects on microbial homeostasis and vector competence.

Vertebrate host-associated microbiota plays a crucial role in mosquito attraction by releasing volatile organic compounds [138]. Takken and Verhulst emphasise that mosquitoes rely on host skin microbiota-derived volatiles, with bacteria such as *Bacillus*, *Brevibacterium*, *Corynebacterium* and *Staphylococcus* producing attractant compounds, while *Pseudomonas* suppresses them [142]. This dynamic interaction suggests a co-evolutionary relationship, where mosquito host-seeking behaviour has been shaped not only by vertebrate hosts but also by their associated microbiomes, reinforcing the role of microbial-mediated chemical communication in mosquito–host interactions. Additionally, Byrd et al. highlight the crosstalk between the immune system and the skin microbiota, demonstrating how resident microorganisms can influence immune responses, promoting either immune tolerance or inflammation [143]. Specific microbial species can strengthen immune homeostasis, while others activate defensive pathways, impacting skin barrier integrity and systemic immunity.

Blood-feeding mosquitoes repeatedly interact with the skin, inoculating saliva during feeding and serving as persistent immune triggers, potentially reshaping local microbial communities and immune responses with each feeding event. Accoti et al. demonstrated that mosquitoes can transmit bacteria present in their saliva into vertebrate hosts during blood feeding [131]. Using fluorescently labelled *S. marcescens*, authors showed that this bacterium was introduced into mice through mosquito bites and later detected in multiple organs, including the liver, lungs, kidneys, spleen, brain and heart. These findings suggest that mosquitoes act as vectors not only for parasites and viruses but also for bacteria, which can be found as part of their microbiota, suggesting a deeper co-evolutionary relationship between mosquitoes, vertebrate hosts and their microbiota. The persistent exposure to mosquito-inoculated microbes may further shape vertebrate immune responses, potentially influencing host susceptibility to infections and immune homeostasis.

The microbiota of mosquitoes and vertebrate hosts acts as a selective force that influences pathogen transmission. Over evolutionary time, selection pressures may have favoured mosquito-associated microbial communities to either facilitate or inhibit pathogen development [144]. Meanwhile, pathogen-induced microbiota shifts of vertebrate hosts could present an adaptive strategy that enhances mosquito attraction. Research has shown that mosquitoes are more attracted to, and more likely to bite, infected hosts rather than uninfected ones. Female mosquitoes can detect the volatile organic compounds (VOCs) produced by host microbiota present in the skin [145].

Environmental factors, such as climate, breeding sites and host diversity, further shape these interactions. Mosquito microbiota is acquired from larval habitats, meaning that ecological disturbances – such as habitat destruction, pollution or climate change – may influence the microbial diversity of mosquito populations. Temporal environmental changes led to variations in the bacterial community structure of mosquitoes, potentially contributing to their physiological changes [146]. Similarly, shifts in vertebrate microbiota may appear owing to dietary changes or environmental stressors, which could further alter mosquito feeding preferences and even reinforce the transmission efficiency of parasites [147, 148]. Understanding how mosquitoes acquire their microbiota and how environmental pressures shape microbial communities is critical for predicting future changes in vector competence and disease dynamics.

The co-evolution of mosquito and vertebrate microbiomes represents a paradigm shift in vector biology. Future research should aim to decipher the long-term evolutionary consequences of microbial exchange, assessing the stability of mosquito microbiota across multiple feeding cycles, and exploring how microbiome-targeted strategies can be effectively integrated into vector control strategies. Innovative approaches that combine genetic manipulation, microbiome engineering, and ecological interventions may provide the next generation of sustainable malaria and vector-borne disease control strategies. The growing recognition of mosquito microbiota as a key determinant of pathogen transmission suggests that holobiont-based strategies could complement existing methods such as insecticide use, genetic modifications and habitat management, offering a more holistic, durable approach to vector control.

In summary, the data in Table 1 emphasise that microbial interactions between holobionts encompass not only direct transfer events but also integrated modulatory processes (environmental, sequential or niche-mediated) that actively contribute to remodelling symbiotic assemblages in host-ectoparasite systems.

### Challenges and future directions

Over the past decade, high-throughput sequencing and advanced bioinformatics tools have revolutionised our understanding of microbial communities, revealing insights into their diversity, ecology, evolution and dynamics [7]. Despite these advancements, significant knowledge gaps remain, as researchers encounter unresolved questions across various studies [10]. For example, what are the functions of key microbial taxa and how do they affect pathogen biology and physiology? How do microbial communities interact with each other and with their host environments? What are the mechanisms by which beneficial microbes suppress or promote pathogen virulence?

While many studies have advanced our understanding of holobiont systems, there is a pressing need for modern computational methods, statistical analysis and advanced bioinformatics to be integrated into future research. In particular, investigating holobiont–holobiont interactions could greatly enhance our understanding of host health, ecological stability and evolutionary dynamics [149].

In both human and veterinary medicine, these innovative approaches have the potential to revolutionise disease prevention, control and diagnostics. Identifying key microbial taxa could lead to development of microbiota-based vaccines, precise microbiome manipulations and targeted interventions to disrupt pathogen transmission [150]. For instance, Mateos-Hernández et al. [45, 46] and Wu-Chuang et al. [47, 48] introduced anti-tick microbiota vaccines by targeting keystone taxa within tick microbial assemblies, thereby reducing pathogen loads transmitted by these blood-sucking ectoparasites. Similarly, Aželytė et al. conducted an initial study of anti-microbiota mosquito vaccine, which aimed to reduce avian malaria infection [90]. Beyond the advantages of anti-microbiota vaccines, microbiome manipulation strategies have the potential to address the growing problem of antibiotic resistance. Microbiome-based therapies could help assess disease susceptibility, manage antibiotic resistance and design treatments that either enhance beneficial microbes or suppress harmful pathogens [150]. One promising para-transgenic strategy is the release of *Wolbachia*-infected *Ae. aegypti*, pioneered by the World Mosquito Program, which demonstrably lowers transmission of DENV, ZIKV, yellow fever and CHIKV; similar symbiont-based modifications of other ectoparasites could open novel avenues for disease control [151].

A promising strategy for controlling vector-borne diseases is the development of immunobiotics – specific bacterial strains that stimulate the immune systems of both hosts and vectors [73]. The goal is to enhance host defences while indirectly influencing vector competence by transferring immune molecules (e.g. cytokines or natural antibodies) during blood feeding of the vector. This cross-species immune activation occurs when host-derived immune molecules interact with the vector’s immune system by binding to receptors, modulating signaling pathways and ultimately affecting pathogen transmission. Rather than providing direct protection to the host, these immune molecules enter the vector and impact the pathogen within its body [73].

Although manipulating the microbiome could alter the holobiont’s immune system, our goal is not to destabilise it. Instead, we seek to modulate microbial interactions to promote pathogen neutralisation, reduce virulence and pathogenicity and influence their ecological translocation into target compartments or niches that favour tropism or facilitate transmission. To this end, manipulation strategies could focus on functional sub-communities within specific ecological niches, allowing for localised, non-disruptive interventions. Microbial taxa that exert neither direct nor indirect influence on pathogen dynamics can thus remain unaltered, preserving the integrity of broader microbial networks and the overall architecture of the holobiont.

Importantly, targeted interventions allow for the preservation of stable or neutral microbial components, ensuring that the integrity of macro-ecological structures remains intact. As holobionts and their associated microbiota evolve (through taxonomic shifts, functional reconfiguration and changing interactions with each other), strategies must be continually updated to maintain their adaptability and ecological viability.

## Conclusions

Unravelling the intricate microbiota–microbiota interactions that occur both within each ectoparasite and host holobiont and across their shared interface represents an emerging and cutting-edge area of research, though it remains insufficiently explored. In this review, we focused on arachnid ectoparasite–host interactions – specifically *Varroa* mites with honeybees and ticks with their vertebrate hosts – and compared them with systems involving non-arachnid ectoparasites, namely bat flies with bats and mosquitoes with vertebrate hosts. The complex interplay between the host organisms and their associated microorganisms forms a dynamic holobiont system that significantly influences the host’s health, behaviour and evolutionary trajectory. Ectoparasites, along with their hosts and microbial communities, constitute a tripartite unit, where each component reciprocally affects the others, highlighting the connection and complexity of these biological systems. An understanding of these complex associations offers considerable potential for microbiota manipulation and infection perturbation, with wide-ranging applications in various sectors such as medicine, agriculture, husbandry and industry. However, fully deciphering the complex relationships among microorganisms, ectoparasites and their hosts requires deeper investigation and novel approaches. Furthermore, it would be advantageous to explore novel insights (e.g. mechanisms of microbial cross-talk and pathways of metabolite exchange) and computational models that are time-efficient and cost-effective, to accelerate the discovery of new sights in this rapidly advancing field. While many ideas and suggestions have been discussed, the aim of this review is not to eradicate parasites from the ecosystem but rather to support hosts in strengthening their resilience to infection. Hosts offer a habitat for parasites, which in turn contribute meaningfully as regulators of ecological diversity.

## Data Availability

Data supporting the main conclusions of this study are included in the manuscript.

## Abbreviations

α-Gal: Galactose-α-1,3-galactose
ALOs: *Arsenophonus*-like organisms
AMPs: Antimicrobial peptides
anti-GluCl: Anti-glutamate-gated chloride channel
DWV: Deformed wing virus
IFN-γ: Interferon *γ*
IGTPase: Inducible guanosine triphosphatase
IMPer: Immunomodulatory peroxidase
MEK-ERK: Mitogen-activated protein kinase kinase–extracellular signal-regulated kinase
PM: Peritrophic matrix
RNAi: Ribonucleic acid interference
SARS: Severe acute respiratory syndrome
STAT: Signal transducer and activator of transcription
TGF-*β*: Transforming growth factor *β*
VOCs: Volatile organic compounds
WHO: World Health Organization

## References

[1] Hassani MA, Durán P, Hacquard S. Microbial interactions within the plant holobiont. Microbiome. 2018;6:58. 29587885 10.1186/s40168-018-0445-0 PMC5870681

[2] Dheilly NM. Holobiont–holobiont interactions: redefining host–parasite interactions. PLoS Pathog. 2014;10:e1004093. 24992663 10.1371/journal.ppat.1004093 PMC4081813

[3] Roughgarden J, Gilbert SF, Rosenberg E, Zilber-Rosenberg I, Lloyd EA. Holobionts as units of selection and a model of their population dynamics and evolution. Biol Theory. 2018;13:44–65.

[4] Garg N. Metabolomics in functional interrogation of individual holobiont members. MSystems. 2021;6:10–1128. 10.1128/mSystems.00841-21 PMC8407464 34427502

[5] Zhang S, Song W, Nothias LF, Couvillion SP, Webster N, Thomas T. Comparative metabolomic analysis reveals shared and unique chemical interactions in sponge holobionts. Microbiome. 2022;10:22. 35105377 10.1186/s40168-021-01220-9 PMC8805237

[6] Williams A. Multiomics data integration, limitations, and prospects to reveal the metabolic activity of the coral holobiont. FEMS Microbiol Ecol. 2024;100:fiae058. 38653719 10.1093/femsec/fiae058 PMC11067971

[7] Hodžić A, Dheilly NM, Cabezas-Cruz A, Berry D. The helminth holobiont: a multidimensional host-parasite-microbiota interaction. Trends Parasitol. 2023;39:91–100. 36503639 10.1016/j.pt.2022.11.012

[8] Alegado RA, Brown LW, Cao S, Dermenjian RK, Zuzow R, Fairclough SR, et al. A bacterial sulfonolipid triggers multicellular development in the closest living relatives of animals. Elife. 2012;1:e00013. 23066504 10.7554/eLife.00013 PMC3463246

[9] Wang X, Zhang A, Miao J, Sun H, Yan G, Wu F, et al. Gut microbiota as important modulator of metabolism in health and disease. RSC Adv. 2018;8:42380–9. 35558413 10.1039/c8ra08094a PMC9092240

[10] Aldana M, Robeva R. New challenges in systems biology: understanding the holobiont. Front Physiol. 2021;12:662878. 33841191 10.3389/fphys.2021.662878 PMC8033030

[11] Koziy RV, Wood SC, Kozii IV, Van Rensburg CJ, Moshynskyy I, Dvylyuk I, et al. Deformed wing virus infection in honey bees (*Apis mellifera* L.). Vet Pathol. 2019;56:636–41. 30857499 10.1177/0300985819834617

[12] Moran NA. Genomics of the honey bee microbiome. Curr Opin Insect Sci. 2015;10:22–8. 26140264 10.1016/j.cois.2015.04.003 PMC4484875

[13] Forsgren E, Olofsson TC, Vásquez A, Fries I. Novel lactic acid bacteria inhibiting *Paenibacillus larvae* in honey bee larvae. Apidologie. 2010;41:99–108.

[14] Zheng H, Nishida A, Kwong WK, Koch H, Engel P, Steele MI, et al. Metabolism of toxic sugars by strains of the bee gut symbiont *Gilliamella apicola*. MBio. 2016;7:e01326–16. 27803186 10.1128/mBio.01326-16 PMC5090037

[15] Zheng H, Powell JE, Steele MI, Dietrich C, Moran NA. Honeybee gut microbiota promotes host weight gain via bacterial metabolism and hormonal signaling. Proc Natl Acad Sci U S A. 2017;114:4775–80. 28420790 10.1073/pnas.1701819114 PMC5422775

[16] Emery O, Schmidt K, Engel P. Immune system stimulation by the gut symbiont *Frischella perrara* in the honey bee (*Apis mellifera*). Mol Ecol. 2017;26:2576–90. 28207182 10.1111/mec.14058

[17] Mead P. Epidemiology of Lyme disease. Infect Dis Clin North Am. 2022;36:495–521. 36116831 10.1016/j.idc.2022.03.004

[18] Kurokawa C, Lynn GE, Pedra JH, Pal U, Narasimhan S, Fikrig E. Interactions between *Borrelia burgdorferi* and ticks. Nat Rev Microbiol. 2020;18:587–600. 32651470 10.1038/s41579-020-0400-5 PMC7351536

[19] Groth M, Skrzydlewska E, Dobrzyńska M, Pancewicz S, Moniuszko-Malinowska A. Redox imbalance and its metabolic consequences in tick-borne diseases. Front Cell Infect Microbiol. 2022;12:870398. 35937690 10.3389/fcimb.2022.870398 PMC9353526

[20] Singh S, Mann B. Insect bite reactions. Indian J Dermatol Venereol Leprol. 2013;79:151–64. 23442453 10.4103/0378-6323.107629

[21] Schön MP. The tick and I: parasite–host interactions between ticks and humans. J Dtsch Dermatol Ges. 2022;20:818–53. 35674196 10.1111/ddg.14821

[22] Glatz M, Means T, Haas J, Steere AC, Müllegger RR. Characterization of the early local immune response to *Ixodes ricinus* tick bites in human skin. Exp Dermatol. 2017;26:263–9. 27623398 10.1111/exd.13207 PMC5342933

[23] Wikel SK. Host immunity to ticks. Annu Rev Entomol. 1996;41:1–22. 8546443 10.1146/annurev.en.41.010196.000245

[24] Talyuli OA, Bottino-Rojas V, Polycarpo CR, Oliveira PL, Paiva-Silva GO. Non-immune traits triggered by blood intake impact vectorial competence. Front Physiol. 2021;12:638033. 33737885 10.3389/fphys.2021.638033 PMC7960658

[25] Al-Khafaji AM, Armstrong SD, Varotto Boccazzi I, Gaiarsa S, Sinha A, Li Z, et al. *Rickettsia buchneri*, symbiont of the deer tick *Ixodes scapularis*, can colonise the salivary glands of its host. Ticks Tick-borne Dis. 2020;11:101299. 31542229 10.1016/j.ttbdis.2019.101299

[26] Gage KL, Schrumpf ME, Karstens RH, Burgdorfer W, Schwan TG. DNA typing of Rickettsiae in naturally infected ticks using a polymerase chain reaction/restriction fragment length polymorphism system. Am J Trop Med Hyg. 1994;50:247–60. 7906924 10.4269/ajtmh.1994.50.247

[27] Baldridge GD, Burkhardt NY, Simser JA, Kurtti TJ, Munderloh UG. Sequence and expression analysis of the *ompA* gene of *Rickettsia peacockii*, an endosymbiont of the rocky mountain wood tick, *Dermacentor andersoni*. Appl Environ Microbiol. 2004;70:6628–36. 15528527 10.1128/AEM.70.11.6628-6636.2004 PMC525201

[28] Breitschwerdt EB, Kordick DL. *Bartonella* infection in animals: carriership, reservoir potential, pathogenicity, and zoonotic potential for human infection. Clin Microbiol Rev. 2000;13:428–38. 10885985 10.1128/cmr.13.3.428-438.2000 PMC88941

[29] McKee CD, Bai Y, Webb CT, Kosoy MY. Bats are key hosts in the radiation of mammal-associated Bartonella bacteria. Infect Genet Evol. 2021;89:104719. 33444855 10.1016/j.meegid.2021.104719 PMC10915969

[30] Wilkinson DA, Duron O, Cordonin C, Gomard Y, Ramasindrazana B, Mavingui P, et al. The bacteriome of bat flies (Nycteribiidae) from the Malagasy region: a community shaped by host ecology, bacterial transmission mode, and host-vector specificity. Appl Environ Microbiol. 2016;82:1778–88. 26746715 10.1128/AEM.03505-15 PMC4784053

[31] Pavanelo DB, Piloto-Sardiñas E, Maitre A, Abuin-Denis L, Kopáček P, Cabezas-Cruz A, et al. Arthropod microbiota: shaping pathogen establishment and enabling control. Front Arachn Sci. 2023;2:1297733.

[32] Duron O, Bouchon D, Boutin S, Bellamy L, Zhou L, Engelstädter J, et al. The diversity of reproductive parasites among arthropods: *Wolbachia* do not walk alone. BMC Biol. 2008;6:27. 18577218 10.1186/1741-7007-6-27 PMC2492848

[33] Xue L, Fang X, Hyman JM. Comparing the effectiveness of different strains of *Wolbachia* for controlling chikungunya, dengue fever, and zika. PLoS Negl Trop Dis. 2018;12:e0006666. 30059498 10.1371/journal.pntd.0006666 PMC6085076

[34] Ogunlade ST, Meehan MT, Adekunle AI, Rojas DP, Adegboye OA, McBryde ES. A review: *Aedes*-borne arboviral infections, controls and *Wolbachia*-based strategies. Vaccines. 2021;9:32. 33435566 10.3390/vaccines9010032 PMC7827552

[35] O’Neal AJ, Singh N, Mendes MT, Pedra JH. The genus Anaplasma: drawing back the curtain on tick–pathogen interactions. Pathog Dis. 2021;79:ftab022. 33792663 10.1093/femspd/ftab022 PMC8062235

[36] Vanderwolf KJ, Campbell LJ, Goldberg TL, Blehert DS, Lorch JM. Skin fungal assemblages of bats vary based on susceptibility to white-nose syndrome. ISME J. 2021;15:909–20. 33149209 10.1038/s41396-020-00821-w PMC8027032

[37] Rosenberg E, Zilber-Rosenberg I. The hologenome concept of evolution after 10 years. Microbiome. 2018;6:78. 29695294 10.1186/s40168-018-0457-9 PMC5922317

[38] Brooks AW, Kohl KD, Brucker RM, van Opstal EJ, Bordenstein SR. Phylosymbiosis: relationships and functional effects of microbial communities across host evolutionary history. PLoS Biol. 2016;14:e2000225. 27861590 10.1371/journal.pbio.2000225 PMC5115861

[39] Kohl KD. Ecological and evolutionary mechanisms underlying patterns of phylosymbiosis in host-associated microbial communities. Philos Trans R Soc Lond B Biol Sci. 2020;375:20190251. 32200746 10.1098/rstb.2019.0251 PMC7133527

[40] Hubert J, Kopecký J, Nesvorna M, Braig HR, Sagova-Mareckova M. Microscopic analysis of the microbiota of three commercial Phytoseiidae species (Acari: Mesostigmata). Exp Appl Acarol. 2017;72:259–71. 10.1007/s10493-020-00520-3. 10.1007/s10493-020-00520-3 PMC7359143 32638184

[41] Wang JQ, Yu T, Qiu HY, Ji SW, Xu ZQ, Cui QC. Differential impact of spotted fever group rickettsia and anaplasmosis on tick microbial ecology: evidence from multi-species comparative microbiome analysis. Front Microbiol. 2025;16:1589263. 40432969 10.3389/fmicb.2025.1589263 PMC12106494

[42] Ruiz-López MJ. Mosquito behavior and vertebrate microbiota interaction: implications for pathogen transmission. Front Microbiol. 2020;11:573371. 33362732 10.3389/fmicb.2020.573371 PMC7755997

[43] Singh BK, Liu H, Trivedi P. Eco-holobiont: a new concept to identify drivers of host-associated microorganisms. Environ Microbiol. 2020;22:564–7. 31849163 10.1111/1462-2920.14900

[44] Silva LM, Acerbi G, Amann M, Koella JC. Exposure to *Pseudomonas* spp. increases *Anopheles gambiae* insecticide resistance in a host-dependent manner. Sci Rep. 2024;14:29789. 39616220 10.1038/s41598-024-78288-4 PMC11608348

[45] Mateos-Hernández L, Obregón D, Maye J, Borneres J, Versille N, De La Fuente J, et al. Anti-tick microbiota vaccine impacts *Ixodes ricinus* performance during feeding. Vaccines. 2020;8:702. 33233316 10.3390/vaccines8040702 PMC7711837

[46] Mateos-Hernández L, Obregón D, Wu-Chuang A, Maye J, Bornères J, Versillé N, et al. Anti-microbiota vaccines modulate the tick microbiome in a taxon-specific manner. Front Immunol. 2021;12:704621. 34322135 10.3389/fimmu.2021.704621 PMC8312226

[47] Wu-Chuang A, Obregon D, Mateos-Hernández L, Cabezas-Cruz A. Anti-tick microbiota vaccines: how can this actually work? Biol. 2022;77:1555–62.

[48] Wu-Chuang A, Mateos-Hernandez L, Maitre A, Rego ROM, Šíma R, Porcelli S, et al. Microbiota perturbation by anti-microbiota vaccine reduces the colonization of *Borrelia afzelii* in *Ixodes ricinus*. Microbiome. 2023;11:151. 37482606 10.1186/s40168-023-01599-7 PMC10364381

[49] Gallai N, Salles J-M, Settele J, Vaissière BE. Economic valuation of the vulnerability of world agriculture confronted with pollinator decline. Ecol Econ. 2009;68:810–21.

[50] Steinhauer NA, Rennich K, Wilson ME, Caron DM, Lengerich EJ, Pettis JS, et al. A national survey of managed honey bee 2012–2013 annual colony losses in the USA: results from the Bee Informed Partnership. J Apic Res. 2014;53:1–18.

[51] Kulhanek K, Steinhauer N, Rennich K, Caron DM, Sagili RR, Pettis JS, et al. A national survey of managed honey bee 2015–2016 annual colony losses in the USA. J Apic Res. 2017;56:328.

[52] Gray A, Adjlane N, Arab A, Ballis A, Brusbardis V, Bugeja Douglas A, et al. Honey bee colony loss rates in 37 countries using the COLOSS survey for winter 2019–2020: the combined effects of operation size, migration and queen replacement. J Apic Res. 2023;62:204–10.

[53] Francis RM, Nielsen SL, Kryger P. *Varroa*–virus interaction in collapsing honey bee colonies. PLoS ONE. 2013;8:e57540. 23526946 10.1371/journal.pone.0057540 PMC3602523

[54] Skičková Š, Svobodová K, Maitre A, Wu-Chuang A, Abuin-Denis L, Piloto-Sardiñas E, et al. Differential impact of *Paenibacillus* infection on the microbiota of *Varroa destructor* and *Apis mellifera*. Heliyon. 2024;10:e39384. 39624306 10.1016/j.heliyon.2024.e39384 PMC11609247

[55] Skičková Š, Kratou M, Svobodová K, Maitre A, Abuin-Denis L, Wu-Chuang A, et al. Functional redundancy and niche specialization in honeybee and *Varroa* microbiomes. Int Microbiol. 2024;28:795–810. 39172274 10.1007/s10123-024-00582-y

[56] Garcia-Gonzalez E, Müller S, Hertlein G, Heid N, Süssmuth RD, Genersch E. Biological effects of paenilamicin, a secondary metabolite antibiotic produced by the honey bee pathogenic bacterium *Paenibacillus larvae*. MicrobiologyOpen. 2014;3:642–56. 25044543 10.1002/mbo3.195 PMC4234257

[57] Wang B, Cheng H, Qian W, Zhao W, Liang C, Liu C, et al. Comparative genome analysis and mining of secondary metabolites of *Paenibacillus polymyxa*. Genes Genet Syst. 2020;95:141–50. 32611933 10.1266/ggs.19-00053

[58] Hertlein G, Seiffert M, Gensel S, Garcia-Gonzalez E, Ebeling J, Skobalj R, et al. Biological role of paenilarvins, iturin-like lipopeptide secondary metabolites produced by the honey bee pathogen Paenibacillus larvae. PLoS ONE. 2016;11:e0164656. 27760211 10.1371/journal.pone.0164656 PMC5070912

[59] Truong A-T, Kang JE, Yoo M-S, Nguyen TT, Youn S-Y, Yoon S-S, et al. Probiotic candidates for controlling *Paenibacillus larvae*, a causative agent of American foulbrood disease in honey bee. BMC Microbiol. 2023;23:150. 37226109 10.1186/s12866-023-02902-0 PMC10207761

[60] Olofsson TC, Butler È, Markowicz P, Lindholm C, Larsson L, Vásquez A. Lactic acid bacterial symbionts in honeybees – an unknown key to honey’s antimicrobial and therapeutic activities. Int Wound J. 2016;13:668–79. 25195876 10.1111/iwj.12345 PMC7949542

[61] Doublet V, Poeschl Y, Gogol-Döring A, Alaux C, Annoscia D, Aurori C, et al. Unity in defence: honeybee workers exhibit conserved molecular responses to diverse pathogens. BMC Genomics. 2017;18:207. 28249569 10.1186/s12864-017-3597-6 PMC5333379

[62] Zanni V, Galbraith DA, Annoscia D, Grozinger CM, Nazzi F. Transcriptional signatures of parasitization and markers of colony decline in *Varroa*-infested honey bees (*Apis mellifera*). Insect Biochem Mol Biol. 2017;87:1–13. 28595898 10.1016/j.ibmb.2017.06.002

[63] Kunc M, Dobeš P, Ward R, Lee S, Čegan R, Dostálková S, et al. Omics-based analysis of honey bee (*Apis mellifera*) response to *Varroa* sp. parasitisation and associated factors reveals changes impairing winter bee generation. Insect Biochem Mol Biol. 2023;152:103877. 36403678 10.1016/j.ibmb.2022.103877

[64] Kuster RD, Boncristiani HF, Rueppell O. Immunogene and viral transcript dynamics during parasitic *Varroa destructor* mite infection of developing honey bee (*Apis mellifera*) pupae. J Exp Biol. 2014;217:1710–8. 24829325 10.1242/jeb.097766

[65] Marche MG, Satta A, Floris I, Pusceddu M, Buffa F, Ruiu L. Quantitative variation in the core bacterial community associated with honey bees from *Varroa-*infested colonies. J Apic Res. 2019;58:444–54.

[66] Kim M, Kim WJ, Park S-J. Analyzing gut microbial community in *Varroa* destructor-infested western honeybee (*Apis mellifera*). J Microbiol Biotechnol. 2023;33:1495–505. 37482801 10.4014/jmb.2306.06040 PMC10699279

[67] Mookhploy W, Krongdang S, Chantawannakul P. Effects of deformed wing virus infection on expressions of immune- and apoptosis-related genes in western honeybees (*Apis mellifera*). Insects. 2021;12:82. 33477797 10.3390/insects12010082 PMC7832323

[68] Kwong WK, Mancenido AL, Moran NA. Immune system stimulation by the native gut microbiota of honey bees. R Soc Open Sci. 2017;4:170003. 28386455 10.1098/rsos.170003 PMC5367273

[69] Kanbar G, Engels W. Ultrastructure and bacterial infection of wounds in honey bee (*Apis mellifera*) pupae punctured by *Varroa* mites. Parasitol Res. 2003;90:349–54. 12684884 10.1007/s00436-003-0827-4

[70] Raymann K, Shaffer Z, Moran NA. Antibiotic exposure perturbs the gut microbiota and elevates mortality in honeybees. PLoS Biol. 2017;15:e2001861. 28291793 10.1371/journal.pbio.2001861 PMC5349420

[71] Schwarz RS, Moran NA, Evans JD. Early gut colonizers shape parasite susceptibility and microbiota composition in honey bee workers. Proc Natl Acad Sci U S A. 2016;113:9345–50. 27482088 10.1073/pnas.1606631113 PMC4995961

[72] Dosch C, Manigk A, Streicher T, Tehel A, Paxton RJ, Tragust S. The gut microbiota can provide viral tolerance in the honey bee. Microorganisms. 2021;9:871. 33920692 10.3390/microorganisms9040871 PMC8072606

[73] Cabezas-Cruz A, Piloto-Sardiñas E, Tonnerre P, Lucas-Torres C, Obregon D. Cross-species immune activation and immunobiotics: a new frontier in vector-borne pathogen control. Trends Parasitol. 2025;41:290–300. 40055101 10.1016/j.pt.2025.02.004

[74] Díaz-Sánchez S, Estrada-Peña A, Cabezas-Cruz A, De La Fuente J. Evolutionary insights into the tick hologenome. Trends Parasitol. 2019;35:725–37. 31331734 10.1016/j.pt.2019.06.014

[75] Bernardo-Cravo AP, Schmeller DS, Chatzinotas A, Vredenburg VT, Loyau A. Environmental factors and host microbiomes shape host-pathogen dynamics. Trends Parasitol. 2020;36:616–33. 32402837 10.1016/j.pt.2020.04.010

[76] Vayssier-Taussat M, Albina E, Citti C, Cosson JF, Jacques MA, Lebrun MH, et al. Shifting the paradigm from pathogens to pathobiome: new concepts in the light of meta-omics. Front Cell Infect Microbiol. 2014. 10.3389/fcimb.2014.00029/abstract. 24634890 10.3389/fcimb.2014.00029 PMC3942874

[77] Bass D, Stentiford GD, Wang H-C, Koskella B, Tyler CR. The pathobiome in animal and plant diseases. Trends Ecol Evol. 2019;34:996–1008. 31522755 10.1016/j.tree.2019.07.012 PMC7479508

[78] Estrada-Peña A, Cabezas-Cruz A, Obregón D. Resistance of tick gut microbiome to anti-tick vaccines, pathogen infection and antimicrobial peptides. Pathogens. 2020;9:309. 32331444 10.3390/pathogens9040309 PMC7238099

[79] Estrada-Peña A, Cabezas-Cruz A, Obregón D. Behind taxonomic variability: the functional redundancy in the tick microbiome. Microorganisms. 2020;8:1829. 33233565 10.3390/microorganisms8111829 PMC7699746

[80] Boulanger N, Insonere J-L-M, Van Blerk S, Barthel C, Serres C, Rais O, et al. Cross-alteration of murine skin and tick microbiome concomitant with pathogen transmission after *Ixodes ricinus* bite. Microbiome. 2023;11:250. 37952001 10.1186/s40168-023-01696-7 PMC10638774

[81] Schommer NN, Gallo RL. Structure and function of the human skin microbiome. Trends Microbiol. 2013;21:660–8. 24238601 10.1016/j.tim.2013.10.001 PMC4744460

[82] Belkaid Y, Tamoutounour S. The influence of skin microorganisms on cutaneous immunity. Nat Rev Immunol. 2016;16:353–66. 27231051 10.1038/nri.2016.48

[83] Chung YW, Gwak HJ, Moon S, Rho M, Ryu JH. Functional dynamics of bacterial species in the mouse gut microbiome revealed by metagenomic and metatranscriptomic analyses. PLoS ONE. 2020;15:e0227886. 31978162 10.1371/journal.pone.0227886 PMC6980644

[84] Sibai M, Altuntaş E, Yıldırım B, Öztürk G, Yıldırım S, Demircan T. Microbiome and longevity: high abundance of longevity-linked Muribaculaceae in the gut of the long-living rodent *Spalax leucodon*. OMICS. 2020;24:592–601. 32907488 10.1089/omi.2020.0116

[85] Liu R, Peng C, Jing D, Xiao Y, Zhu W, Zhao S, et al. *Lachnospira* is a signature of antihistamine efficacy in chronic spontaneous urticaria. Exp Dermatol. 2022;31:242–7. 34558729 10.1111/exd.14460

[86] Hawlena H, Rynkiewicz E, Toh E, Alfred A, Durden LA, Hastriter MW, et al. The arthropod, but not the vertebrate host or its environment, dictates bacterial community composition of fleas and ticks. ISME J. 2013;7:221–3. 22739493 10.1038/ismej.2012.71 PMC3526175

[87] Rynkiewicz EC, Hemmerich C, Rusch DB, Fuqua C, Clay K. Concordance of bacterial communities of two tick species and blood of their shared rodent host. Mol Ecol. 2015;24:2566–79. 25847197 10.1111/mec.13187

[88] Zolnik CP, Prill RJ, Falco RC, Daniels TJ, Kolokotronis S. Microbiome changes through ontogeny of a tick pathogen vector. Mol Ecol. 2016;25:4963–77. 27588381 10.1111/mec.13832

[89] Landesman WJ, Mulder K, Allan BF, Bashor LA, Keesing F, LoGiudice K, et al. Potential effects of blood meal host on bacterial community composition in *Ixodes scapularis* nymphs. Ticks Tick Borne Dis. 2019;10:523–7. 30660375 10.1016/j.ttbdis.2019.01.002 PMC6441379

[90] Aželytė J, Wu-Chuang A, Žiegytė R, Platonova E, Mateos-Hernandez L, Maye J, et al. Anti-microbiota vaccine reduces avian malaria infection within mosquito vectors. Front Immunol. 2022;13:841835. 35309317 10.3389/fimmu.2022.841835 PMC8928750

[91] Smith AA, Navasa N, Yang X, Wilder CN, Buyuktanir O, Marques A, et al. Cross-species interferon signaling boosts microbicidal activity within the tick vector. Cell Host Microbe. 2016;20:91–8. 27374407 10.1016/j.chom.2016.06.001 PMC4945435

[92] Surachetpong W, Singh N, Cheung KW, Luckhart S. MAPK ERK signaling regulates the TGF-β1-dependent mosquito response to *Plasmodium falciparum*. PLoS Pathog. 2009;5:e1000366. 19343212 10.1371/journal.ppat.1000366 PMC2658807

[93] Samantsidis GR, Kwon H, Wendland M, Fonder C, Smith RC. TNF signaling mediates cellular immune function and promotes malaria parasite killing in the mosquito *Anopheles gambiae*. PLoS Pathog. 2024. 10.1101/2024.05.02.592209. 10.1371/journal.ppat.1013329 PMC12244535 40608824

[94] Cano-Argüelles AL, Piloto-Sardiñas E, Maitre A, Mateos-Hernández L, Maye J, Wu-Chuang A, et al. Microbiota-driven vaccination in soft ticks: implications for survival, fitness and reproductive capabilities in *Ornithodoros moubata*. Mol Ecol. 2024;33:e17506. 39161118 10.1111/mec.17506

[95] Obregón D, Bard E, Abrial D, Estrada-Peña A, Cabezas-Cruz A. Sex-specific linkages between taxonomic and functional profiles of tick gut microbiomes. Front Cell Infect Microbiol. 2019;9:298. 31475121 10.3389/fcimb.2019.00298 PMC6702836

[96] Gough JM, Kemp DH. Localization of a low abundance membrane protein (Bm86) on the gut cells of the cattle tick *Boophilus microplus* by immunogold labeling. J Parasitol. 1993;79:900–7. 8277383

[97] Willadsen P. Novel vaccines for ectoparasites. Vet Parasitol. 1997;71:209–22. 9261979 10.1016/s0304-4017(97)00028-9

[98] Belperron AA, Bockenstedt LK. Natural antibody affects survival of the spirochete *Borrelia burgdorferi* within feeding ticks. Infect Immun. 2001;69:6456–62. 11553590 10.1128/IAI.69.10.6456-6462.2001 PMC98781

[99] Maitre A, Wu-Chuang A, Aželytė J, Palinauskas V, Mateos-Hernández L, Obregon D, et al. Vector microbiota manipulation by host antibodies: the forgotten strategy to develop transmission-blocking vaccines. Parasit Vectors. 2022;15:4. 34983601 10.1186/s13071-021-05122-5 PMC8725291

[100] Kumar M, Kaur S, Kariu T, Yang X, Bossis I, Anderson JF, et al. *Borrelia burgdorferi* BBA52 is a potential target for transmission blocking Lyme disease vaccine. Vaccine. 2011;29:9012–9. 21945261 10.1016/j.vaccine.2011.09.035 PMC3202674

[101] Dobson AP. What links bats to emerging infectious diseases? Science. 2005;310:628–9. 16254175 10.1126/science.1120872

[102] Calisher CH, Childs JE, Field HE, Holmes KV, Schountz T. Bats: important reservoir hosts of emerging viruses. Clin Microbiol Rev. 2006;19:531–45. 16847084 10.1128/CMR.00017-06 PMC1539106

[103] Reeves WK, Beck J, Orlova MV, Daly JL, Pippin K, Revan F, et al. Ecology of bats, their ectoparasites, and associated pathogens on Saint Kitts Island. J Med Entomol. 2016;53:1218–25. 27282816 10.1093/jme/tjw078

[104] Szentiványi T, Christe P, Glaizot O. Bat flies and their microparasites: current knowledge and distribution. Front Vet Sci. 2019;6:115. 31106212 10.3389/fvets.2019.00115 PMC6492627

[105] Dick CW, Dittmar K. Parasitic bat flies (Diptera: Streblidae and Nycteribiidae): host specificity and potential as vectors. In: Klimpel S, Mehlhorn H, editors. Bats (Chiroptera) as Vectors of Diseases and Parasites. Heidelberg: Springer; 2014.

[106] Dick CW, Patterson BD. Bat flies: obligate ectoparasites of bats. In: Morand S, Krasnov BR, Poulin R, editors. Micromammals and Macroparasites [Internet]. Tokyo: Springer; 2006.

[107] Herrera GL, Gutierrez E, Hobson KA, Altube B, Díaz WG, Sánchez-Cordero V. Sources of assimilated protein in five species of New World frugivorous bats. Oecologia. 2002;133:280–7. 28466224 10.1007/s00442-002-1036-z

[108] Fenton BM, Simmons N. Bats: a world of science and mystery. Chicago: The University of Chicago Press; 2014.

[109] Bai Y, Osinubi MOV, Osikowicz L, McKee C, Vora NM, Rizzo MR, et al. Human exposure to novel *Bartonella* species from contact with fruit bats. Emerg Infect Dis. 2018;24:2317–23. 30457529 10.3201/eid2412.181204 PMC6256376

[110] Sakoui S, Derdak R, Addoum B, Serrano-Delgado A, Soukri A, El Khalfi B. The life hidden inside caves: ecological and economic importance of bat guano. Int J Ecol. 2020;2020:2020:7.

[111] Veikkolainen V, Vesterinen EJ, Lilley TM, Pulliainen AT. Bats as reservoir hosts of human bacterial pathogen, *Bartonella mayotimonensis*. Emerg Infect Dis. 2014;20:960–7. 24856523 10.3201/eid2006.130956 PMC4036794

[112] Boulouis H-J, Chao-chin C, Henn JB, Kasten RW, Chomel BB. Factors associated with the rapid emergence of zoonotic *Bartonella* infections. Vet Res. 2005;36:383–410. 15845231 10.1051/vetres:2005009

[113] Spach DH, Koehler JE. Bartonella-associated infections. Infect Dis Clin North Am. 1998;12:137–55. 9494835 10.1016/s0891-5520(05)70414-1

[114] Urushadze L, Bai Y, Osikowicz L, McKee C, Sidamonidze K, Putkaradze D, et al. Prevalence, diversity, and host associations of Bartonella strains in bats from Georgia (Caucasus). PLoS Negl Trop Dis. 2017;11:e0005428. 28399125 10.1371/journal.pntd.0005428 PMC5400274

[115] Davoust B, Marié J-L, Dahmani M, Berenger J-M, Bompar J-M, Blanchet D, et al. Evidence of *Bartonella* spp. in blood and ticks (*ornithodoros hasei*) of bats, in French Guiana. Vector Borne Zoonotic Dis. 2016;16:516–9. 27305604 10.1089/vbz.2015.1918

[116] Loftis AD, Gill JS, Schriefer ME, Levin ML, Eremeeva ME, Gilchrist MJR, et al. Detection of *Rickettsia*, *Borrelia*, and *Bartonella* in *Carios kelleyi* (Acari: Argasidae). J Med Entomol. 2005;42:473–80. 15962801 10.1093/jmedent/42.3.473

[117] Banyard AC, Hayman DTS, Freuling CM, Müller T, Fooks AR, Johnson N. Bat rabies rabies: scientific basis of the disease and its management [Internet]. Cambridge: Academic Press; 2013.

[118] Leroy EM, Kumulungui B, Pourrut X, Rouquet P, Hassanin A, Yaba P, et al. Fruit bats as reservoirs of Ebola virus. Nature. 2005;438:575–6. 16319873 10.1038/438575a

[119] Epstein JH, Field HE, Luby S, Pulliam JRC, Daszak P. Nipah virus: impact, origins, and causes of emergence. Curr Infect Dis Rep. 2006;8:59–65. 16448602 10.1007/s11908-006-0036-2 PMC7088631

[120] Banerjee A, Kulcsar K, Misra V, Frieman M, Mossman K. Bats and coronaviruses. Viruses. 2019;11:41. 30634396 10.3390/v11010041 PMC6356540

[121] Wang L-F, Shi Z, Zhang S, Field H, Daszak P, Eaton B. Review of bats and SARS. Emerg Infect Dis. 2006;12:1834–40. 17326933 10.3201/eid1212.060401 PMC3291347

[122] Zhao H. COVID-19 drives new threat to bats in China. Science. 2020;367:1436–1436. 32217719 10.1126/science.abb3088

[123] Xu Z, Feng Y, Chen X, Shi M, Fu S, Yang W, et al. Virome of bat-infesting arthropods: highly divergent viruses in different vectors. J Virol. 2022;96:e0146421. 34586860 10.1128/jvi.01464-21 PMC8865543

[124] Moreira LA, Iturbe-Ormaetxe I, Jeffery JA, Lu G, Pyke AT, Hedges LM, et al. A *Wolbachia* symbiont in *Aedes aegypti* limits infection with dengue, chikungunya, and *Plasmodium*. Cell. 2009;139:1268–78. 20064373 10.1016/j.cell.2009.11.042

[125] Simón F, González-Miguel J, Diosdado A, Gómez PJ, Morchón R, Kartashev V. The complexity of zoonotic filariasis episystem and its consequences: a multidisciplinary view. Biomed Res Int. 2017;2017:6436130. 28642878 10.1155/2017/6436130 PMC5469992

[126] WHO. World malaria report 2023. Geneva: World Health Organization; 2023.

[127] Romoli O, Gendrin M. The tripartite interactions between the mosquito, its microbiota and *Plasmodium*. Parasit Vectors. 2018;11:200. 29558973 10.1186/s13071-018-2784-x PMC5861617

[128] Guégan M, Zouache K, Démichel C, Minard G, Tran Van V, Potier P, et al. The mosquito holobiont: fresh insight into mosquito-microbiota interactions. Microbiome. 2018;6:49. 29554951 10.1186/s40168-018-0435-2 PMC5859429

[129] Romoli O, Schönbeck JC, Hapfelmeier S, Gendrin M. Production of germ-free mosquitoes via transient colonisation allows stage-specific investigation of host–microbiota interactions. Nat Commun. 2021;12:942. 33574256 10.1038/s41467-021-21195-3 PMC7878806

[130] Mancini MV, Damiani C, Accoti A, Tallarita M, Nunzi E, Cappelli A, et al. Estimating bacteria diversity in different organs of nine species of mosquito by next generation sequencing. BMC Microbiol. 2018;18:126. 30286722 10.1186/s12866-018-1266-9 PMC6172810

[131] Accoti A, Damiani C, Nunzi E, Cappelli A, Iacomelli G, Monacchia G, et al. Anopheline mosquito saliva contains bacteria that are transferred to a mammalian host through blood feeding. Front Microbiol. 2023;14:1157613. 37533823 10.3389/fmicb.2023.1157613 PMC10392944

[132] Onyango MG, Payne AF, Stout J, Dieme C, Kuo L, Kramer LD, et al. *Aedes albopictus* saliva contains a richer microbial community than the midgut. Parasit Vectors. 2024;17:267. 38918848 10.1186/s13071-024-06334-1 PMC11197185

[133] Devenport M, Fujioka H, Jacobs-Lorena M. Storage and secretion of the peritrophic matrix protein Ag-Aper1 and trypsin in the midgut of *Anopheles gambiae*. Insect Mol Biol. 2004;13:349–58. 15271206 10.1111/j.0962-1075.2004.00488.x

[134] Kumar S, Molina-Cruz A, Gupta L, Rodrigues J, Barillas-Mury C. A peroxidase/dual oxidase system modulates midgut epithelial immunity in *Anopheles gambiae*. Science. 2010;327:1644–8. 20223948 10.1126/science.1184008 PMC3510679

[135] Hatfield PR. Detection and localization of antibody ingested with a mosquito bloodmeal. Med Vet Entomol. 1988;2:339–45. 2980192 10.1111/j.1365-2915.1988.tb00206.x

[136] Vaughan JA, Azad AF. Passage of host immunoglobulin G from blood meal into hemolymph of selected mosquito species (Diptera: Culicidae). J Med Entomol. 1988;25:472–4. 2905008 10.1093/jmedent/25.6.472

[137] Meyers JI, Gray M, Foy BD. Mosquitocidal properties of IgG targeting the glutamate-gated chloride channel in three mosquito disease vectors (Diptera: Culicidae). J Exp Biol. 2015;218:1487–95. 25994632 10.1242/jeb.118596 PMC4448666

[138] Shi H, Yu X, Cheng G. Impact of the microbiome on mosquito-borne diseases. Protein Cell. 2023;14:743–61. 37186167 10.1093/procel/pwad021 PMC10599646

[139] Lutz M, Knaus P. Integration of the TGF-*β* pathway into the cellular signalling network. Cell Signal. 2002;14:977–88. 12359303 10.1016/s0898-6568(02)00058-x

[140] Luckhart S, Crampton AL, Zamora R, Lieber MJ, Dos Santos PC, Peterson TML, et al. Mammalian transforming growth factor *β*1 activated after ingestion by *Anopheles stephensi* modulates mosquito immunity. Infect Immun. 2003;71:3000–9. 12761076 10.1128/IAI.71.6.3000-3009.2003 PMC155698

[141] Cappelli A, Damiani C, Mancini MV, Valzano M, Rossi P, Serrao A, et al. *Asaia* activates immune genes in mosquito eliciting an anti-*Plasmodium* response: implications in malaria control. Front Genet. 2019;10:836. 31608103 10.3389/fgene.2019.00836 PMC6774264

[142] Takken W, Verhulst NO. Chemical signaling in mosquito–host interactions: the role of human skin microbiota. Curr Opin Insect Sci. 2017;20:68–74. 28602238 10.1016/j.cois.2017.03.011

[143] Byrd AL, Belkaid Y, Segre JA. The human skin microbiome. Nat Rev Microbiol. 2018;16:143–55. 29332945 10.1038/nrmicro.2017.157

[144] Boissière A, Tchioffo MT, Bachar D, Abate L, Marie A, Nsango SE, et al. Midgut microbiota of the malaria mosquito vector Anopheles gambiae and interactions with Plasmodium falciparum infection. PLoS Pathog. 2012;8:e1002742. 22693451 10.1371/journal.ppat.1002742 PMC3364955

[145] Busula AO, Verhulst NO, Bousema T, Takken W, De Boer JG. Mechanisms of *Plasmodium*-enhanced attraction of mosquito vectors. Trends Parasitol. 2017;33:961–73. 28942108 10.1016/j.pt.2017.08.010

[146] Tchioffo MT, Boissière A, Abate L, Nsango SE, Bayibéki AN, Awono-Ambéné PH, et al. Dynamics of bacterial community composition in the malaria mosquito’s epithelia. Front. 2016. 10.3389/fmicb.2015.01500/abstract. 10.3389/fmicb.2015.01500 PMC4700937 26779155

[147] Videvall E, Marzal A, Magallanes S, Fleischer RC, Espinoza K, García-Longoria L. The uropygial gland microbiome of house sparrows with malaria infection. J Avian Biol. 2021. 10.1111/jav.02686.

[148] De Boer JG, Robinson A, Powers SJ, Burgers SLGE, Caulfield JC, Birkett MA, et al. Odours of *Plasmodium falciparum*-infected participants influence mosquito–host interactions. Sci Rep. 2017;7:9283. 28839251 10.1038/s41598-017-08978-9 PMC5570919

[149] Matthews JL. Editorial: holobiont interactions. Front Ecol Evol. 2024;12:1382169.

[150] Greer R, Dong X, Morgun A, Shulzhenko N. Investigating a holobiont: microbiota perturbations and transkingdom networks. Gut Microbes. 2016;7:126–35. 26979110 10.1080/19490976.2015.1128625 PMC4856449

[151] World Mosquito Program. (2025). Reducing mosquito-borne diseases with *Wolbachia*. Available from: https://www.worldmosquitoprogram.org

